# Evolution of *Fusarium tricinctum* and *Fusarium avenaceum* mitochondrial genomes is driven by mobility of introns and of a new type of palindromic microsatellite repeats

**DOI:** 10.1186/s12864-020-6770-2

**Published:** 2020-05-12

**Authors:** Nadia Ponts, Charlotte Gautier, Jérôme Gouzy, Laetitia Pinson-Gadais, Marie Foulongne-Oriol, Christine Ducos, Florence Richard-Forget, Jean-Michel Savoie, Chen Zhao, Gérard Barroso

**Affiliations:** 1 INRAE, MycSA, F-33882 Villenave d’Ornon, France; 2 LIPM, Université de Toulouse, INRAE, CNRS, Castanet-Tolosan, France; 3 Academy of National Food and Strategic Reserves Administration, Beijing, China; 4 University of Bordeaux, INRAE, MycSA, F-33882 Villenave d’Ornon, France

**Keywords:** Group I intron, Homing endonuclease, Lateral transfer, Palindrome, *Fusarium tricinctum* species complex

## Abstract

**Background:**

Increased contamination of European and Asian wheat and barley crops with “emerging” mycotoxins such as enniatins or beauvericin, produced by *Fusarium avenaceum* and *Fusarium tricinctum,* suggest that these phylogenetically close species could be involved in future food-safety crises.

**Results:**

The mitochondrial genomes of *F. tricinctum* strain INRA104 and *F. avenaceum* strain FaLH27 have been annotated. A comparative analysis was carried out then extended to a set of 25 wild strains. Results show that they constitute two distinct species, easily distinguished by their mitochondrial sequences. The mitochondrial genetic variability is mainly located within the intergenic regions. Marks of variations show they have evolved (i) by Single Nucleotide Polymorphisms (SNPs), (ii) by length variations mediated by insertion/deletion sequences (Indels), and (iii) by length mutations generated by DNA sliding events occurring in mononucleotide (A)_n_ or (T)_n_ microsatellite type sequences arranged in a peculiar palindromic organization. The optionality of these palindromes between both species argues for their mobility. The presence of Indels and SNPs in palindrome neighbouring regions suggests their involvement in these observed variations. Moreover, the intraspecific and interspecific variations in the presence/absence of group I introns suggest a high mobility, resulting from several events of gain and loss during short evolution periods. Phylogenetic analyses of intron orthologous sequences suggest that most introns could have originated from lateral transfers from phylogenetically close or distant species belonging to various Ascomycota genera and even to the Basidiomycota fungal division.

**Conclusions:**

Mitochondrial genome evolution between *F. tricinctum* and *F. avenaceum* is mostly driven by two types of mobile genetic elements, implicated in genome polymorphism. The first one is represented by group I introns. Indeed, both genomes harbour optional (inter- or intra-specifically) group I introns, all carrying putatively functional *heg*s, arguing for a high mobility of these introns during short evolution periods. The gain events were shown to involve, for most of them, lateral transfers between phylogenetically distant species. This study has also revealed a new type of mobile genetic element constituted by a palindromic arrangement of (A) n and (T) n microsatellite sequences whose presence was related to occurrence of SNPs and Indels in the neighbouring regions.

## Background

Fusarium Head Blight (FHB) is a fungal disease of cereals caused by infection of grains by various (up to 15) *Fusarium* species [1]. Among these *Fusarium* species, five are commonly associated in cereal crops grown in Europe: *Fusarium graminearum*, *Fusarium culmorum*, *Fusarium avenaceum*, *Fusarium tricinctum* and *Fusarium poae* [2]. These five species are all able to produce mycotoxins that accumulate in grains and contaminate processed food and feed products, representing both a health risk and an important economic stake. Several of these mycotoxins (e.g.*,* deoxynivalenol and zearalenone) are targeted by European and international regulations fixing maximum admissible levels in food and feeds (regulation EC 2006/2009). Others, such as fusaproliferin, moniliformin, enniatins and beauvericin, are increasingly found as crop contaminants and considered as “emerging” [3]. Recent toxicological studies suggested enniatins could be genotoxic and hepatotoxic [4], and could be involved in future food-safety crises, especially in a context of climate and agricultural practices changes. As an example, a three-year study (2011–2013) on 11 malting barley varieties cultivated in Italy revealed changes in the *Fusarium* species constituting the FHB complex depending on the variety used and/or specific weather parameters occurring during the seasons, the enniatin-producers *F. avenaceum* and *Fusarium tricinctum* being consistently present [1]. This study confirmed previous experiments of wheat ears co-inoculation by two different Fusarium species [5] which have shown that an increase of wetness period and temperature led to an increase of the FHB symptoms and of the mycotoxin (trichothecene) productivity (up to 1000 times). Similarly, numerous studies reported increased frequencies of contamination of European and Asian wheat and barley crops with enniatins mainly produced by *F. avenaceum* and *F. tricinctum* [2, 6, 7]*.* In a recent study, Orlando et al. [8] analyzed enniatin-contents and contaminations with *Fusarium* species in 1240 samples of small grain cereals (wheat, durum wheat, spring barley, triticale and winter barley) from 2012 to 2014 French harvests, and found enniatins produced by *F. avenaceum* and *F. tricinctum* highly prevalent in French small grain cereals at levels consistently at their highest on spring barley (mean values of 199 to 1316 μg/kg).

In the past decade, phylogenetic studies as well as fungal genetic and genomic approaches have led to an important increase in the number of species identified as belonging to the *Fusarium* monophyletic genus [9]. Among them, *F. avenaceum* (Fries) [10] and *F. tricinctum* (Corda) [11] are considered as two closely related taxa ranged in the same *F. tricinctum* species complex [12]. In this *Fusarium tricinctum* species complex (FTSC), *Fusarium acuminatum* and *Fusarium arthrosporioides* [13, 14] appear also closely related to both species, whereas *Fusarium torulosum*, *Fusarium flocciferum* and *Fusarium petersiae* are grouped in a more distant sister clade [12, 15]. In any case, the scarcity of data on both the genetic diversity of these fungal phytopathogens and the biosynthesis and regulation pathways of their associated mycotoxins limits our ability to assess the toxinogenic risk they represent, and consequently to design appropriate responses. In this context, the complete genome sequence of *F. tricinctum* strain INRA104 has recently been obtained in a whole genome shotgun project, including its full-length gapless mitochondrial genome [16]. In the present study, we investigated the modalities of molecular evolution of mitochondrial genomes in the *Fusarium* genus. We annotated the mitochondrial genome (MtDNA) of *F. tricinctum* strain INRA104 and compared its molecular organization with that of previously sequenced mitochondrial genomes of *F. avenaceum* [17] as well as of other distant *Fusarium* species [18, 19]. Then, to study the phylogenetic relationship between these closely related species and assessing the accuracy of mitochondrial sequences to discriminate *F. tricinctum* and *F. avenaceum*, the polymorphic mitochondrial regions were characterized and their intra- and inter- specific variability was studied in a set of 25 previously assigned wild strains. The mitochondrial polymorphism was compared with that of two nuclear genes *rpb1* and *rpb2*, previously reported as discriminating markers of both species [12]. The mobility and origin of all the harboured mitochondrial group I introns was also studied.

## Results

### Molecular organisation and phylogenetic analysis of the mitochondrial genomes

From our whole genome sequencing project (DDB/ENA/GenBank accession number QFZF00000000), the sequence of the complete mitochondrial genome (GenBank accession number CM009895) of *F. tricinctum* strain INRA104 has been retrieved and annotated (FtriMtDNA). In parallel, the mitochondrial genome of *F. avenaceum* strain FaLH27 (GenBank accession number JQGE01000002.1), a phylogenetically closely related species, was retrieved from third party sequencing data produced by Lysøe et al. [17] and also annotated for the purpose of comparison. The mitochondrial genomes of *F. avenaceum* strain FaLH27 (FaveMtDNA) and *F. tricinctum* strain INRA 104 have a size of 49,396 bp and 48,506 bp, respectively and the same average GC-content of 33%. A second *F. avenaceum* annotated mitochondrial genome sequence was also found available in the GenBank for strain FaLH03 (49,402 bp, accession number JQGD01000004.1). It differs from FaveMtDNA strain FaLH27 by only 6 bp in length, and possesses more than 99.9% of nucleotide identity. To avoid redundancy, the sequence of the MtDNA of the FaLH03 strain was not included in this study. *F. tricinctum* strain INRA104 and *F. avenaceum* strain FaLH27 MtDNAs are close in size, FaLH27 MtDNA being 890 bp longer (representing 1.8% of variation in length scattered along the whole mitochondrial genome), and show high sequence conservation with 98.9% nucleotide identity.

**Fig. 1:**
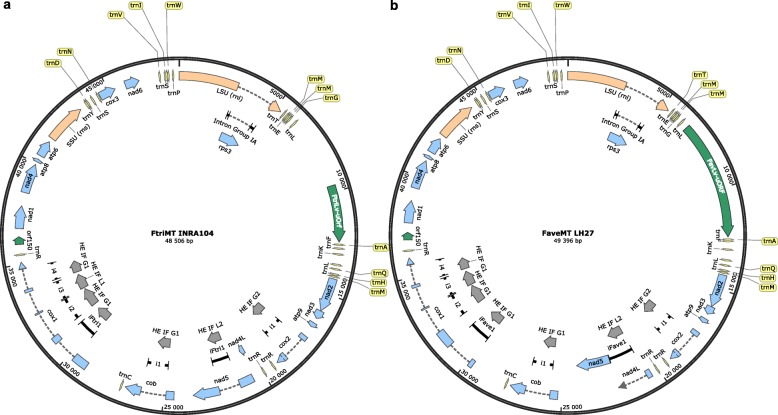
Physical map of *F. tricinctum* strain INRA104 (**a**) and *F. avenaceum* strain FaLH27 (**b**) mitochondrial genomes. Nucleotide 1 is arbitrarily set as the first nucleotide of LSU (rnl). Intron sequences are indicated by thick black lines. Within introns sequences, the carried ORF encoding putatively functional homing endonuclease (HE) have been represented; all are in frame (IF) with the upstream exon; the HE family is also indicated (G1, G2, L1 and L2 represent homing endonuclease characterized by one or two GIY-YIG motif(s), one or two LAGLIDADG motif(s), respectively)

Annotation results of MtDNAs from both *F. tricinctum* strain INRA104 and *F. avenaceum* strain FaLH27 are displayed in Fig. 1, Additional file 1 (GenBank formatted annotated sequence of the *F. tricinctum* strain INRA104 MtDNA) and Additional file 2 (GenBank formatted annotated sequence of the *F. avenaceum* strain FaLH27 MtDNA). For easier comparison of the molecular organization of the *F. tricinctum* and *F. avenaceum* MtDNAs with others previously annotated MtDNAs in the fungal kingdom [20] and especially in the *Fusarium* genus, including those reported by Al-Reedy et al. [18], both sequences were annotated by arbitrarily fixing the first nucleotide as the 5′ end of the *rnl* gene encoding the large ribosomal subunit RNA. This first nucleotide corresponds to positions 40,818 bp in *F. tricinctum* INRA 104 MtDNA and 21,516 bp in *F. avenaceum* FaLH27 one (GenBank accessions CM009895 and JQGE01000002, respectively).

**Fig. 2:**
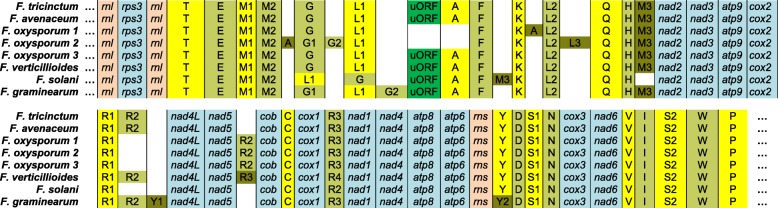
Schematic representation of gene synteny in *Fusarium* mitochondrial genomes. Annotations for *F. oxysporum*, *F. graminearum* and *F. solani* were retrieved from Al-Reedy et al. [16] and Brankovics et al. [17]. *F. oxysporum 1, 2* and *3* are the three variants of the large variable region characterized in Brankovics et al. [17], strains Fon015, FOSC3-a, and NRRL37622, respectively. Annotations for *F. tricinctum* strain INRA104 and *F. avenaceum* strain FaLH27 are from the present study. rDNA genes *rnl* and *rns* are in light orange; typical mitochondrial protein genes are in pale blue; tRNAs in yellow to olive green shades; uORFs are in bright green

*F. tricinctum* and *F. avenaceum* MtDNAs possess the same set of mitochondrial structural genes than all the *Fusarium* mitochondrial genomes reported to date [18]. Fourteen typical mitochondrial genes encode subunits of the electron transport chain and of the ATP-synthase complex. They include seven subunits of the electron transport complex I (*nad1, 2, 3, 4, 4 L, 5* and *6*), one subunit of complex III (*cob*), three subunits of complex IV (*cox1, 2* and *3*) and three subunits of the F_0_ ATP-synthase complex (*atp6, 8* and *9*). *Fusarium* mitochondrial coding sequences (CDS) show a conserved synteny (Fig. 2). All genes are indeed located on the same strand and share the same order. Notably, in all analysed *Fusarium* species, *nad2* and *nad3* genes are joined (no intergenic sequence), and *nad4*L and *nad5* genes are fused, i.e.*,* the last nucleotide of the *nad4* termination codon TAA is also the first nucleotide of the *nad5* ATG initiation codon (Fig. 1 and Additional files 1 and 2). Regarding structural RNA genes, *F. tricinctum* and *F. avenaceum* MtDNAs possess the LSU and SSU rDNAs and the same set of 26 tRNAs, strictly identical in sequences, including one tRNA “Sup” type (anticodon TCA) that reads the tryptophane (W) codon TGA. This is the only tRNA coding the amino-acid W carried by these genomes. Among the genes encoding mitochondrial proteins, it is to be noted that the gene *rps3* encoding a protein involved in the assembly of the mitochondrial ribosome is located in the intron of LSU-rDNA, a feature shared by all *Fusarium* MtDNAs reported to date, and also by a large number of filamentous and yeast Ascomycota [20, 21]. The only variability observed in synteny concerns the presence/absence of several tRNAs, or their relocation in other intergenic regions of the genome (Fig. 2). For example, additional tRNAs have been described in *F. oxysporum* variant 2 (tRNAs G2 and L3), *F. verticillioides* (tRNA R3), *F. solani* (tRNA M3), and *F. graminearum* (tRNA G2 and Y1). The tRNAs A or R2 are relocated in all displayed *F. oxysporum* variants, and the order of the tRNAs G and L1 is inverted in *F. solani* (versus the other *Fusarium spp.*). tRNA genes occupy seven intergenic regions of the *F. tricinctum* and *F. avenaceum* MtDNAs in which they are either alone, by pairs, or grouped by four to seven (Figs. 1 and 2).

**Fig. 3:**
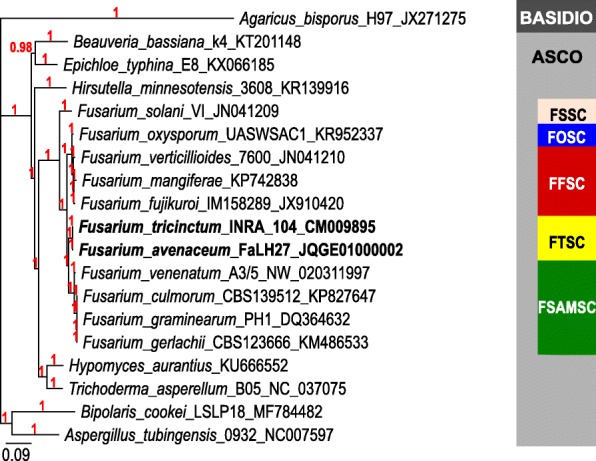
Unrooted phylogenetic tree of *Fusarium* species and of distant or closely related *Ascomycota* species based on compiled complete CDS sequence alignment of the 14 typical mitochondrial genes (*nad2, nad3, atp9, cox2, nad4L, nad5, cob, cox1, nad1, nad4, atp8, atp6, cox3, nad6*). Posterior probabilities (Bayesian inference; 1,000,000 generations) are indicated in red. Labels show species names followed by the name and/or number of the strain and the GenBank accession number corresponding to the sequence used. Basidio = Basidiomycota; Asco = Ascomycota; FSSC = *Fusarium solani* Species Complex; FSAMSC = *Fusarium sambucinum* Species Complex; FTSC = *Fusarium tricinctum* Species Complex; FOSC = *Fusarium oxysporum* Species Complex; FFSC = *Fusarium fujikuroi* Species Complex

Phylogenetic relationship was inferred from the 14 mitochondrial CDS sequences (all mitochondrial structural genes *nad1* to *6*, *cob*, *cox1 to 3*, *atp6, 8* and 9), concatenated, between 11 *Fusarium* species belonging to five species complexes (FSSC, FSAMSC, FTSC, FOSC, FFSC), seven distant Ascomycota and one Basidiomycota (Fig. 3). The size of the compiled sequences varied from 11,721 bp (for the Basidiomycota *Agaricus bisporus*) to 14,769 bp (for the Ascomycota *Epichloe typhina*); from 12,878 bp (*F. mangiferae*) and 13,144 bp (*F. fujikuroi*) for species of the *Fusarium* genus. This tree is fully congruent with those reported in previous phylogenetic studies based on the nuclear markers *rpb1* and *rpb2* [12]. In this mitochondrial CDS-based tree, the FTSC appears distant from the Fusarium sambucinum Species Complex (FSAMSC) as well as the related Fusarium oxysporum (FOSC) and Fusarium fujikuroi (FFSC) Species Complexes. As previously reported [12], the *Fusarium solani* Species Complex (FSSC) ranges in an outgroup position in regard of the other *Fusarium* species complexes. For comparison, a phylogenetic tree based on the *cox1* CDS (from 1587 bp to 1593 bp in all the available *Fusarium* species) has also been built (Additional file 3: Fig. S1). This gene has been chosen because it is known to possess the highest number of group I introns in the fungal kingdom and in the *Fusarium* genus [22], and consequently can also be used to evidence conflict in phylogenetic trees based on orthologous intronic sequences of mitochondrial genes. Both trees based on the 14 compiled CDS and on the *cox1* CDS are fully congruent, showing the lack of gene conflict between *cox1* exonic sequences and all other mitochondrial CDS from a phylogenetic point of view.

### Location of polymorphic versus conserved features

The analysis of the overall molecular organisation of *F. tricinctum* and *F. avenaceum* MtDNAs allowed distinguishing two remarkable regions: (*i*) a large region constituted by a long unidentified open reading frame flanked by two tRNAs clusters, previously named as Lv-uORF (for large variable unidentified ORF) in other Fusaria by Al-Reedy et al. [18]; (*ii*) the rest of the genome containing all conserved typical mitochondrial genes (39,586 bp and 40,439 bp in length in *F. tricinctum* and *F. avenaceum,* respectively). In *F. avenaceum*, the uORF included in this 8956 bp-long region (from nt 5290 to nt 14,245) is 5865 bp in size (from nt 6503 to nt 12,367). This ORF is reduced in *F. tricinctum* strain INRA104 which harbours an eroded uORF of only 2748 bp corresponding to the 3’end of the *F. avenaceum* one (for a total variable region of 8919 bp in length in *F. tricinctum*, spanning from 5261 bp to 14,179 bp).

CDS encoding mitochondrial proteins of both *F. tricinctum* and *F. avenaceum* were found highly conserved in sequence and length. For example, COX1 CDS sequences (1593 bp) of both species differ by only three SNPs (99.8% of identity). When comparing with other species of the *Fusarium* genus, percentages of identity with *F. tricinctum* COX1 CDS varied between 92.5% for the distant *F. solani*, and 94–96% for members of the FOSC, FFSC or FSAMSC which includes *F. graminearum*.

As a whole, comparison of the mitochondrial genome of *F. tricinctum* (48,506 bp) with that of *F. avenaceum* (49,396 bp) revealed a global mutation frequency of 7.83 events/kb explained by 314 SNPs (6.47 SNP/kb) and 66 Indels (1.36 Indel/kb). The total size of these Indel sequences represented 1713 bp, i.e.*,* 3.5% of the *F. tricinctum* genome size (see Additional file 3: Table S1 and Table S2 for details). Although coding sequences (rDNA, tRNA, and protein-coding CDS) represent 43.8% of the total *F. tricinctum* mitochondrial genome, they contain less than 8% of all 380 mutations events (30 SNPs, or 1.41 SNP/kb). With 1.63 SNP/kb and 2.21 Indels/kb, the introns found in these structural genes are only slightly more polymorphic, mostly due to the presence of Indels (absent from coding sequences). Most of the genetic variability (mainly composed of SNPs) is found in intergenic regions and in the large variable uORF-including region, which concentrate 47.9 and 39.2% of the mutation events in 22.1 and 16.4% of the genome, respectively (or 16.95 and 18.76 total mutation events/kb, respectively).

**Fig. 4:**
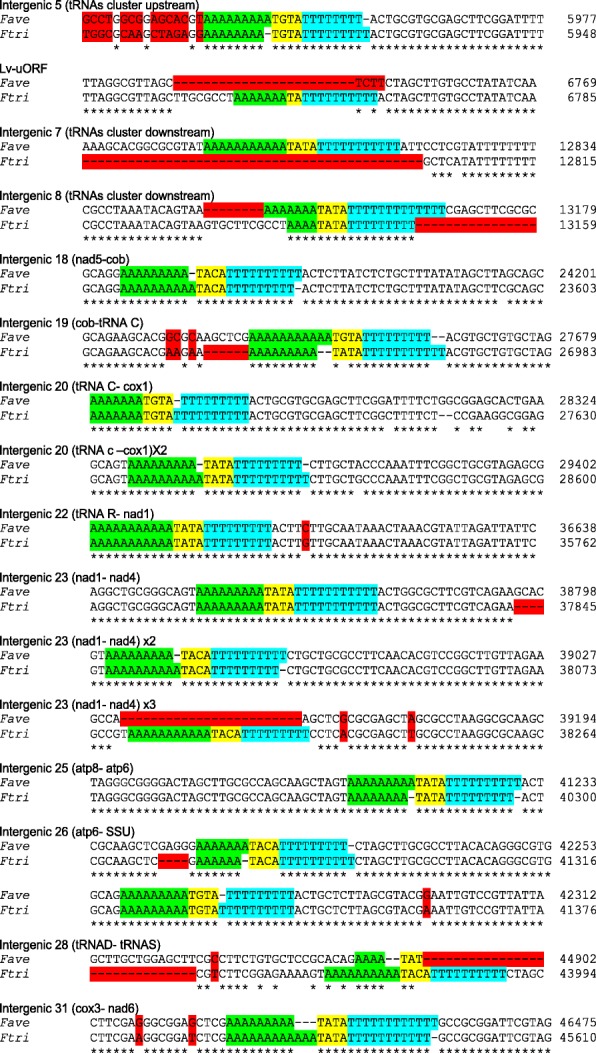
Alignment of mitochondrial intergenic sequences from *F. tricinctum* strain INRA104 et *F. avenaceum* strain FaLH27 containing polymorphic microsatellite sequences arranged in a palindromic organisation. The (A)_n_ microsatellite type repetitions are in green, the (T)_n_ microsatellite type repetitions are in blue, separated by spacer sequences (from two to generally four nucleotides) in yellow. SNP and Indels leading to polymorphic regions and located near or within microsatellite sequences are highlighted in red

Strikingly, seven intergenic regions (N°18, 19, 20, 23, 25, 26 and 31) were shown to be affected by length variations occurring in microsatellites regions with a palindromic type organization (Fig. 4). Indeed, these regions are composed of (A)_7–12_-TATA (or TGTA or TACA or TA)-(T)_9–11_ sequences. Beside these polymorphic regions, this peculiar palindromic organization of mononucleotide microsatellites was found 14 and 16 times in *F. avenaceum* and *F. tricinctum* MtDNA sequences, respectively. In the details, palindromes were found in the polymorphic regions listed above, and also in both tRNAs clusters flanking the Lv-uORF as well as in the intergenic regions N°22 and 28 (Fig. 4). When comparing both species, three palindromes were absent from FaveMtDNA (in the Lv-uORF and the intergenic regions 23 and 28) and one from FtriMtDNA (in the intergenic region 7). Moreover, these palindromes were frequently found associated with large deletions or sequences variations in the neighbouring regions (Fig. 4, lv-uORF, and intergenic regions 5, 7, 8, 19, 26 and 28).

### Characterisation of mitochondrial introns and lv-uORF of *F. tricinctum* strain INRA104 and *F. avenaceum* strain FaLH27

MtDNAs of *F. tricinctum* strain INRA104 and *F. avenaceum* strain FaLH27 were shown to each harbour eight group I introns. Among them, the LSU-rDNA intron (group IA) containing the ribosomal protein *rps3* gene is present in both *F. tricinctum* and *F. avenaceum,* as it is the case for all the mitochondrial genomes described to date in *Fusarium* species [18] and in most of the Ascomycota filamentous species [20, 21]. Regarding mitochondrial protein structural genes, five introns were present in both species: one in *cox2* (*cox2 i1*), one in *cob* (*cob i1*), and three in *cox1* (*cox1 i2*, *cox1 i3* and *cox1 i4*). Each of these five introns was assumed to represent orthologous sequences on the basis of a shared insertion site in the CDS of the gene as well as a high nucleotide sequence identity (> 99%). In addition to these five conserved introns, *F. tricinctum* possesses one intron in *nad5 (nad5 iFtri1*) and one intron in the 5′ part of *cox1* (*cox1 iFtri1*) that are not present in *F. avenaceum*; in the same way, *F. avenaceum* MtDNA harbours an intron in *nad4*L (*nad4L iFave1*) that is not present in *F. tricinctum* as well as an intron located in the 5′ part of *cox1* (*cox1 iFave1*) but different from *cox1 iFtri1* in sequence (only 49.3% of nucleotide sequence identity) and in its insertion site. All these group I introns have a size between 1018 bp *(cox1 i4*) and 1481 bp (*nad4L iFave1*), and all carry an intact ORF in frame with the upstream exon which encodes a potentially functional homing endonuclease (HE) acting in the homing (site-specific integration) of the intron in the corresponding CDS after lateral transfer [23].

For each of these nine group I introns, their optionality (presence/absence pattern) was investigated in a panel of 25 strains constituted by 14 *F. tricinctum* and 11 *F. avenaceum* strains (Table 1 and Additional file 3: Table S3) by a fragment-length PCR approach that uses primers defined in exons flanking the investigated intron (Additional file 3: Table S4). Results, expressed by the presence or absence of each intron, were obtained for the 14 *F. tricinctum* and 11 *F. avenaceum* studied strains and compiled in Table 1. Among the five introns shared by *F. tricinctum* strain INRA104 and *F. avenaceum* strain FaLH27 – i.e.*, cox2 i1, cob i1, cox1 i2, cox1 i3,* and *cox1 i4* – the three introns located in the 3′ part of the *cox1* gene (*cox1 i2*, *i3* and *i4*) were the only ones present in all the 25 studied strains. The intron *cox2 i1* was found in all but one strain, *F. avenaceum* INRA612. The intron *cob i1* was missing in 9 out of 14 of the *F. tricinctum* strains. Considering the two introns found in *F. tricinctum* INRA104 and not in *F. avenaceum* FaLH27 – i.e.*, cox1 iFtri1* and *nad5 iFtri1* – *cox1 iFtri1* was indeed present in all investigated *F. tricinctum* strains while absent from all *F. avenaceum* ones whereas *nad5 iFtri1* was not exclusive to *F. tricinctum* but also found in two out of 11 *F. avenaceum* strains (*F. avenaceum* INRA6 and *F. avenaceum* INRA612). Sequencing of corresponding PCR products showed high nucleotide identity (99.5%, i.e.*,* five SNP on the whole 1.025 kb-long intron sequence). Regarding the two introns found in *F. avenaceum* FaLH27 but not in *F. tricinctum* INRA104 – i.e.*, nad4L iFave1* and *cox1 iFave1*, *nad4L iFave1* was found in all *F. avenaceum* strains but not in any of the investigated *F. tricinctum* strains, whereas *cox1 iFave1* was present in all *F. avenaceum* strains but also in one *F. tricinctum* strain (INRA86). As a whole, 9 out of 11 of the *F. avenaceum* studied strains have identical intronic pattern. Two strains, *F. avenaceum* INRA6 and *F. avenaceum* INRA612, nonetheless differ by the presence of the *nad5 iFtri1*, and the lack of *cox2 i1* for strain INRA612. In the 14 *F. tricinctum* studied strains, two groups can be distinguished based on the absence or presence of *cob i1*, herein referred to as group A (nine strains) and group B (five strains), respectively. In group B, only strain INRA86 shows a specific intronic profile characterized by the additional presence of *cox1 iFave1*.

**Table 1:** Optionality of *F. tricinctum* and *F. avenaceum* mitochondrial introns

***Species***	***Strain***	*cox2 i1*	*nad4L iFave1*	*nad5 iFtri1*	*cob i1*	*cox1 ifave1*	*cox1 iFtri1*	*cox1 i2*	*cox1 i3*	*cox1 i4*
*F. tricinctum*	INRA104	**P**^**a**^	**Exon**	**P**	**P**	**Exon**	**P**	**P**	**P**	**P**
*F. tricinctum*	INRA 105	**P**	**Exon**	**P**	**P**	**Exon**	**P**	**P**	**P**	**P**
*F. tricinctum*	INRA 106	**P**	**Exon**	**P**	**P**	**Exon**	**P**	**P**	**P**	**P**
*F. tricinctum*	INRA 610	**P**	**Exon**	**P**	**P**	**Exon**	**P**	**P**	**P**	**P**
*F. tricinctum*	INRA 521	**P**	**Exon**	**P**	**Exon**	**Exon**	**P**	**P**	**P**	**P**
*F. tricinctum*	INRA 522	**P**	**Exon**	**P**	**Exon**	**Exon**	**P**	**P**	**P**	**P**
*F. tricinctum*	INRA 523	**P**	**Exon**	**P**	**Exon**	**Exon**	**P**	**P**	**P**	**P**
*F. tricinctum*	INRA 524	**P**	**Exon**	**P**	**Exon**	**Exon**	**P**	**P**	**P**	**P**
*F. tricinctum*	INRA 525	**P**	**Exon**	**P**	**Exon**	**Exon**	**P**	**P**	**P**	**P**
*F. tricinctum*	INRA 526	**P**	**Exon**	**P**	**Exon**	**Exon**	**P**	**P**	**P**	**P**
*F. tricinctum*	INRA 527	**P**	**Exon**	**P**	**Exon**	**Exon**	**P**	**P**	**P**	**P**
*F. tricinctum*	INRA 528	**P**	**Exon**	**P**	**Exon**	**Exon**	**P**	**P**	**P**	**P**
*F. tricinctum*	INRA 529	**P**	**Exon**	**P**	**Exon**	**Exon**	**P**	**P**	**P**	**P**
*F. tricinctum*	INRA 86	**P**	**Exon**	**P**	**P**	**P**	**P**	**P**	**P**	**P**
*F. avenaceum*	FaLH27	**P**	**P**	**Exon**	**P**	**P**	**Exon**	**P**	**P**	**P**
*F. avenaceum*	INRA 112	**P**	**P**	**Exon**	**P**	**P**	**Exon**	**P**	**P**	**P**
*F. avenaceum*	INRA 494	**P**	**P**	**Exon**	**P**	**P**	**Exon**	**P**	**P**	**P**
*F. avenaceum*	INRA 495	**P**	**P**	**Exon**	**P**	**P**	**Exon**	**P**	**P**	**P**
*F. avenaceum*	INRA 496	**P**	**P**	**Exon**	**P**	**P**	**Exon**	**P**	**P**	**P**
*F. avenaceum*	INRA 497	**P**	**P**	**Exon**	**P**	**P**	**Exon**	**P**	**P**	**P**
*F. avenaceum*	INRA 498	**P**	**P**	**Exon**	**P**	**P**	**Exon**	**P**	**P**	**P**
*F. avenaceum*	INRA 499	**P**	**P**	**Exon**	**P**	**P**	**Exon**	**P**	**P**	**P**
*F. avenaceum*	INRA 611	**P**	**P**	**Exon**	**P**	**P**	**Exon**	**P**	**P**	**P**
*F. avenaceum*	INRA 6	**P**	**P**	**P**	**P**	**P**	**Exon**	**P**	**P**	**P**
*F. avenaceum*	INRA 612	**Exon**	**P**	**P**	**P**	**P**	**Exon**	**P**	**P**	**P**

^a^Present

**Fig. 5:**
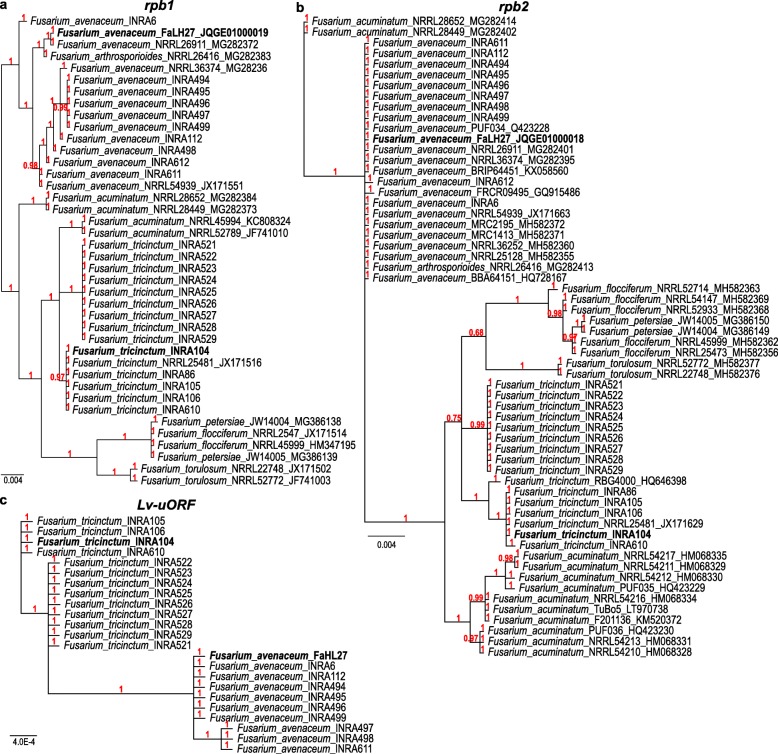
Unrooted phylogenetic trees of strains belonging to the *Fusarium tricinctum* species complex (FTSC), including strains of the species *F. tricinctum*, *F. avenaceum*, *F. acuminatum, F. arthrosporioides*, *F. torulosun*, *F. flocciferum*, and *F. petersiae*. Trees are based on the nuclear markers *rpb1* partial sequences (798 bp) (**a**), *rpb2* partial sequence (762 bp) (**b**) or a part of the mitochondrial Lv-uORF (818 bp) (**c**). Posterior probabilities (Bayesian inference; 1,000,000 generations) are indicated in red. Species name is followed by the name of the strain and accession numbers when sequences were retrieved from Genbank

For comparison purposes, we categorize the 25 strains of this study using as marker the partial sequences of PCR products derived from *rpb1* (798 bp), *rpb2* (762 bp) and a part of the mitochondrial Lv-uORF (818 bp) (Fig. 5a, b and c, respectively). For each marker, all orthologous sequences of strains and species belonging to the FTSC and available in the GenBank (see Additional file 4 Table S6 for accession numbers) have been added to the analyses (i.e.*,* 40 *rpb1* sequences and 60 *rpb2* sequences in total; see Fig. 5a and b). The trees obtained with nuclear sequences are highly congruent, showing that *F. tricinctum* and *F. avenaceum* strains separate well from each other as well as from the other species included in this analysis, with the exception of *F. arthrosporioides* NRRL26416 that clusters with *F. avenaceum* strains. All other species were separated in a phylogenetically distant clade. Regarding *F. acuminatum*, the studied strains seem to cluster in a sister group of the *F. tricinctum* clade except for *F. acuminatum* NRRL28652 and NRRL28449 that regroup with *F. avenaceum* strains. These results suggest that *F. acuminatum* may be more closely related to *F. tricinctum* than *F. avenaceum*, strains NRRL28652 and NRRL28449 being exceptions possibly belonging to the *F. avenaceum* or a new undescribed species. Strikingly, the two clades separated on the base of the presence/absence of *cob i1* described earlier, match the clear separation of *F. tricinctum* strains in two groups in the *rpb1* and *rpb2*-based trees (Fig. 5a and b, respectively).

A similar analysis was performed using the mitochondrial sequences of the large variable intergenic region carrying the uORF only (Fig. 5c). The obtained tree clearly separated *F. tricinctum* and *F. avenaceum* into two clades but fails to discriminate most strains of the same species. Indeed, nucleotide identities vary from 99.9 to 100% between strains of the same species, and less than 97.6% between strains of both species. On the sequence alignment (Additional file 3: Fig. S2), two insertion/deletion events can be observed: a deletion of three nucleotides in *F. tricinctum* vs. *F. avenaceum* at the beginning of the alignment, and an insertion of 39 to 45 nucleotides. These events are responsible for the erosion of the uORF in the *F. tricinctum* strains. These Indels are accompanied by several flanking interspecific SNPs. Strikingly, the large *F. tricinctum* inserted sequence is bordered by a copy of a polymorphic microsatellite region with the peculiar organization described earlier, i.e.*,* (A)_7_ –TA-(T)_9_–_10_.

### Phylogenetic analyses of *F. tricinctum* and *F. avenaceum* mitochondrial group I introns

To explore the origin and evolution history of each of these mitochondrial introns, orthologous sequences available in the GenBank have been compiled (see Additional file 4 Table S6 for accession numbers). Intron sequences were considered orthologous when they were members of the same Position class (PCl), as previously defined [22]: each PCl contains intron sequences possessing both the highest percentage of nucleotide identity and the same location in the exonic sequence (CDS). Results are summarized in Table 2.

**Table 2:** Summary of mitochondrial intron distribution, and origin and mobility hypotheses

*A* Mitochondrial intron distribution in fungal strains and percentage of nucleotide identity between orthologous sequences								
**Intron**	**Presence**							
	**In*F. tricinctum*clade**^**a**^	**In*F. avenaceum*clade**^**a**^	**In FTSC**	**In FSAMSC**	**In FOSC**	**In FFSC**	**In FSSC**	**In distant Ascomycota**
***cox2 i1***	**all**	**all but INRA 612**	**optional**	***Fcul, Fven, Fgra*(95.7–96.2%)**	**0**	**0**	**0**	***N. cinnabarina*(84.2%)**
***nad4L iFave1***	**0**	**all**	**optional**	***Fcul, Fven*(91.7–96.9%)**	**0**	**0**	**0**	***T. asperellum*(97.7%);*H. minnesotensis*(81.7%)**
***nad5 iFtri1***	**all**	**2 (INRA6, INRA 612)**	**optional**	**0**	**0**	**0**	**0**	**Pleosporineae sub-order (84.1–85.6%)**
***cob i1***	**5 strains (clade B)**	**all**	**optional**	***Fven*(97.9%),*Fgra, Fger*(89.7%)**	**several*Foxy f.sp.***	**0**	**0**	***B. bassiana*(81.9%)**
***cox1 iFave1***	**only INRA86**	**all**	**optional**	***Fcul, Fven, Fgra, Fger*(94.5%)**	**0**	***Fman (95.1%), Ftem*(93,7%)**	**0**	***E. typhina*(84.2%)**
***cox1 iFtri1***	**all**	**0**	**optional**	***Fcul, Fven, Fgra, Fger*(80.6%)**	**0**	**0**	***Fsol*(94.4%)**	***A. aegerita*(85.4%); Basidiomycota**
***cox1 i2***	**all**	**all**	**all**	***Fcul, Fven, Fgra, Fger*(77.7%)**	**0**	***Fver, Ftem, Fcir*(77.2%)**	***Fsol*(93.0%)**	**0**
***cox1 i3***	**all**	**all**	**all**	***Fcul, Fven, Fgra, Fger*(66.5%)**	**0**	***Ftem, Fcir*(66.5%)**	***Fsol*(96.0%)**	***Atub*(86.6%)**
***cox1 i4***	**all**	**all**	**all**	***Fcul, Fven, Fgra, Fger*(96.5%)**	**0**	***Ftem, Fcir*(74.5%)**	**0**	**0**
*B* Origin and mobility hypotheses for mitochondrial introns								
**Intron**		**Mobility (presence in*Fusarium*genus)**		**Origin hypothesis**		**Lateral transfer**		
***cox2 i1***		**Yes (lost in INRA 612)**		**ancestral (in*Fusarium*genus)**		**unknown**		
***nad4L iFave1***		**Yes (frequent loss)**		**ancestral (in*Fusarium*genus)**		**Yes (recent transfer with*Tasp*)**		
***nad5 iFtri1***		**yes (rare intron; frequent loss)**		**ancestral (in FTSC)**		**unknown**		
***cob i1***		**Yes (loss in one*Ftri*clade)**		**ancestral (in*Fusarium*genus)**		**Yes (recent transfer with*Fven*)**		
***cox1 iFave1***		**Yes (loss and gain)**		**ancestral (in*Fusarium*genus)**		**Yes (recent transfer with*Fman*)**		
***cox1 iFtri1***		**Yes (loss in*Fave*)**		**ancestral (in*Fusarium*genus)**		**Yes (recent transfer with*Fsol*)**		
***cox1 i2***		**Yes (loss and gain in*Fusarium*genus)**		**ancestral (in*Fusarium*genus)**		**Yes (recent transfer with*Fsol*)**		
***cox1 i3***		**Yes (loss and gain in*Fusarium*genus)**		**ancestral (in*Fusarium*genus)**		**Yes (recent transfer with*Atub*)**		
***cox1 i4***		**Yes (loss and gain in*Fusarium*genus)**		**ancestral (in*Fusarium*genus)**		**unknown**		

^**a**^Presence of the introns in the 14 *F. tricinctum* and 11 *F. avenaceum* studied strains; when applicable, ranges of percentage of nucleotide identity with species outside of *F. tricinctum* and *F. avenaceum* are given in parenthesis; *FculF. culmorum*, *FvenF. venenatum*, *FgraF. graminearum*, *FgerF. gerlachii*, *FoxyF. oxysporum*, *FmanF. mangiferae*, *FtemF. temperatum*, *FcirF. circinatum*, *FsolF. solani*, *AtubAspergillus tubigensis*, *TaspTrichoderma asperellum*

Among the nine introns reported in *F. tricinctum* and *F. avenaceum* mitochondrial genes, only *nad5 iFtri1* did not possess any orthologous sequences in other *Fusarium* species. For this intron, only few orthologous sequences described in five Ascomycota species were found, all belonging to the Pleosporinae suborder, with 84.1 to 85.6% nucleotide identity: *Bipolaris cookei* and *Bipolaris maydis*, *Leptosphaeria biglobosa* ‘brassicae’, *Leptosphaeria maculans* ‘lepidii’, and *Coniothyrium glycines*.

Orthologous sequences of the eight remaining introns were found in other *Fusarium* species complexes, mainly in members of the FSAMSC. The *cob i1* intron was shown to also possess orthologous sequences in members of the FOSC, whereas the five introns carried by *cox1* had orthologous sequences in members of the FFSC with the exception of *cox1 iFtri1*. Orthologous sequences of *cox1 iFtri1* as well as *cox1 i2* and *cox1 i3* were found in members of the FSSC. Notably, percentages of nucleotide identity between the various orthologous sequences included in this study were highly variable, arguing for different origins and evolution of the intronic sequences.

**Fig. 6:**
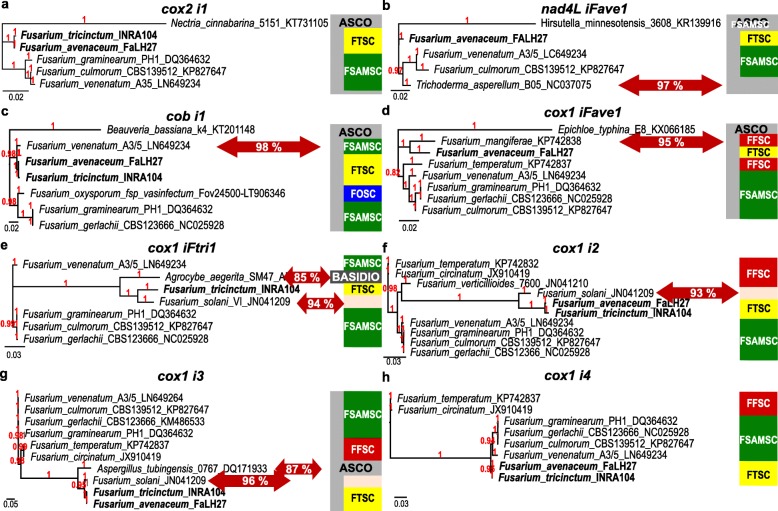
Unrooted phylogenetic trees of *Fusarium spp.* strains based on the sequences of orthologous mitochondrial introns of *F. tricinctum* and/or *F. avenaceum cox2 i1* (**a**), *nad4L iFave1* (**b**), *cob i1* (**c**), *cox1 iFave1* (**d**), *cox1 iFtri1* (**e**), *cox1 i2* (**f**), *cox1 i3* (**g**), *cox1 i4* (**h**). Posterior probabilities (Bayesian inference; 1,000,000 generations) are indicated in red. Labels show species names followed by the name and/or number of the strain and the GenBank accession number corresponding to the sequence used. Basidio = Basidiomycota; Asco = Ascomycota; FSSC = *Fusarium solani* Species Complex; FSAMSC = *Fusarium sambucinum* Species Complex; FTSC = *Fusarium tricinctum* Species Complex; FOSC = *Fusarium oxysporum* Species Complex; FFSC = *Fusarium fujikuroi* Species Complex. Red arrows indicate species involved in a lateral transfer with one member or ancestor of the FTSC; percentages of nucleotide identity between the corresponding orthologous sequences are indicated on the arrows

Phylogenetic trees were built based on the alignments of the orthologous sequences of each intron (Fig. 6). Whilst species of the FSAMSC, FTSC, and FFSC are well separated from each other and from other Ascomycota in the trees built from *cox2 i1* and *cox1 i4* orthologous sequences (Fig. 6a and h, respectively), the Ascomycota *Trichoderma asperellum* is inserted among *Fusarium* species of the FSAMSC when *nad4L iFave1* is considered (Fig. 6b), with 97% nucleotide identity, suggesting a lateral transfer event. Similarly, at least five other lateral transfer events can be observed regarding *cob1 i1* (involving *Fusarium venenatum,* Fig. 6c*), cox1 iFave1* (involving *Fusarium mangiferae,* Fig. 6d*), cox1 iFtri1* (involving the distant Basidiomycota *Agrocybe aegerita,* Fig. 6e*), cox i3* (involving *Aspergillus tubingensis,* Fig. 6g), as well as three introns of *cox1* (*cox1 iFtri1*, *cox1 i2* and *cox1 i3*) and involving *F. solani* (Fig. 6e, f and g).

## Discussion

The mitochondrial genomes of *F. tricinctum* strain INRA104 (48.5 kb) and *F. avenaceum* strain FaLH27 (49.4 kb) were found highly similar in size and sequence (98.3% nucleotide identity), with an identical GC content (33%). The comparative analysis of their molecular organization identified a variable region with high interspecific polymorphism, comprising an ORF with cryptic function (uORF of 5865 nt, 1955 aa) flanked by two clusters of tRNAs, the ORF being eroded (2748 nt, 916 aa) in *F. tricinctum* strain INRA104 by comparison with the very large one found in *F. avenaceum* FaLH27. This region was previously defined by Al-Reedy et al. [18] as an “exceptionally large ORF” in *F. verticillioides* (2572 aa), *F. solani* (2013 aa) and *F. graminearum* (1931 aa). In a recent report based on the analysis of 61 mitochondrial genomes of the FOSC, Brankovics et al. [19] described a large uORF of 2284 aa in some strains or eroded smaller ORFs (less than 740 aa) in other strains, similarly to our observations in *F. tricinctum* and *F. avenaceum*. Here, we found that both the large variable uORF-including regions and intergenic regions exhibit similar patterns of frequent mutation events, thus arguing for the optionality of this large uORF. Searching nuclear genomes of uORF-devoid species/strains for the presence of paralogous sequences may provide elements towards understanding the evolution history of this optional ORF of cryptic function.

Surprisingly, we found that Lv-uORFs identified in *F. tricinctum* strain INRA104 and *F. avenaceum* FaLH27 as well as their flanking tRNAs clusters (in the intergenic regions 5 and 7) harboured microsatellite sequences (A)_n_ and (T)_n_ arranged in a palindrome-type organisation, and were also affected by large Indels and/or important sequence variations. Such association between this peculiar palindromic organisation and Indels was also found in several other intergenic regions of *F. tricinctum* and *F. avenaceum* mitochondrial genomes. The optionality of these palindromes between *F. tricinctum* and *F. avenaceum* argues for their mobility, as previously described for mitochondrial double-hairpin elements of *Allomyces* fungi [24]. Moreover, the frequent presence of large Indels and/or important sequence variations in the neighbouring regions of the palindromes of *F. tricinctum* and *F. avenaceum* suggests the involvement of this new type of putative mobile genetic elements in the observed deletions, and sequence evolution. Such repetitive palindromic repeats were found up to 19 times in the genome of the green microalga *Lobosphaera incisa* [25] and have been proposed to act as substrates for large-scale genomic rearrangements or as sites of transcript processing. In the same way, hairpin and tandem repeat-containing genetic elements, and also tRNAs with associated palindromic sequences have been described in cryptophyte algae, which possess a clear phylogenetic relationship to heterotrophic eukaryotes [26]. In these algae species, these sequences are believed to be involved in the observed gene shuffling affecting mitochondrial genes by promoting recombination events.

In fungal MtDNAs, dispersed repeated sequences have already been reported in filamentous or yeast Ascomycota, such as *Pst*1 (GC-rich) palindromes in *Neurospora crassa* [27, 28], the mitochondrial ultra-short element MUSE1 (11 bp in size, more than 100 copies per genome) in *Podospora anserina* [29], or GC clusters in *Saccharomyces cerevisiae* and 28 other yeast species [30]. Various roles have been proposed for such fungal interspersed elements, such as sites of initiation for replication or transcription, or processing of transcripts, but most of them are considered as mobile genetic elements, invasive and highly recombinogenic, and involved in mitochondrial polymorphism by generation of length mutations (excision, insertion, DNA slippage) [27–30].

To the best of our knowledge, the palindromic organisation of (A) n and (T) n microsatellites sequences described in the *F. tricinctum* and *F. avenaceum* MtDNA constitute the first report of such putatively mobile (AT-rich) genetic elements. To date, nothing is known about their putative role (e.g.*,* in the molecular evolution of mitochondrial genomes, the MtDNA replication or the regulation of the expression of genes). However, our results by showing a frequent link between these palindromes and the occurrence of SNP and/or Indels in their neighbouring regions (see Fig. 4), suggest a putative role of these mobile elements in the generation of mitochondrial polymorphism. Notably, despite the high number (14 to 16) of these palindromic sequences in FtriMtDNA and FaveMtDNA, no evidence of gene shuffling or recombination event was recovered.

When compared with other mitochondrial genomes available in the whole monophyletic genus *Fusarium*, the sizes of *F. tricinctum* and *F. avenaceum* MtDNAs are close to the sizes reported for 61 strains of the FOSC, ranging between 38.7 kb in *F. oxysporum f. sp. melonis* and 53.4 kb in *F. oxysporum f. sp. lycopersici* [19], as well as for *F. verticillioides* (53.7 kb) of the FFSC [18]. Other species have larger MtDNAs, such as *F. solani* (63 kb, [18]) a member of the FSSC located at the base of the phylogenetic tree of the *Fusarium* genus [12]. Species of the FSAMSC have considerably larger MtDNAs [31, 32], in the range of 93.4 kp for *Fusarium gerlachii* (GenBank accession number KM486533) to 103.8 kb for *F. culmorum* (GenBank Accession number KP827647). As previously reported in numerous fungal genera [22], the mitochondrial genome size variations observed in the *Fusarium* genus does not involve the highly conserved set of mitochondrial genes and is not related to the phylogenetic distances separating these *Fusarium* species. Here, size variation would be mainly due to variations in the group I intron content of these genomes. For a still unknown reason, some species seem to behave as large intron reservoirs whereas other closely related ones show few or none introns [22].

Group I introns are mobile genetic elements that can be gained (after lateral transfers, even between phylogenetically distant species) or lost, during short evolution periods [22, 33]. The identification of putatively functional Homing Endonucleases genes (*hegs*) in all introns of *F. tricintum* and *F. avenaceum* mitochondrial genomes supports that the concerned introns are independent functional mobile genetic elements with homing specificity. Comparing the molecular organization of MtDNAs of *F. tricinctum* and *F. avenaceum*, we found significant polymorphisms between these closely related species in relation with intron mobility. This inter- or intra-specific optionality can be explained by two divergent hypotheses [22, 33–35]: (*i*) a gain of the mobile genetic element after a lateral transfer between a phylogenetically close or distant species [35, 36] or (*ii*) the loss of an ancestrally inherited genetic element [34, 36]. We explored the origin and evolution history of the introns found in *F. tricinctum* and *F. avenaceum*, and searched, for each intron, its available orthologous sequences to compare their phylogenetic relationships to the one established using the compiled typical mitochondrial protein genes in close and distant Ascomycota species (Fig. 3 and Table 2). Among the nine introns characterized in the *F. tricinctum* and/or *F. avenaceum* studied strains, eight were found widely distributed in the *Fusarium* genus, including in members of other *Fusarium* species complexes. Only the intron *nad5 iFtri1* was found solely in strains belonging to the FTSC, precisely in all the 14 studied *F. tricinctum* strains plus 2 *F. avenaceum* strains out of 11 studied, suggesting that this intron is a rare intron ancestrally inherited in the FTSC. This specificity suggests that its evolution history in the Ascomycota phylum would be intron loss rather than gain and spreading.

The patchy distribution of the eight other introns in the *Fusarium* genus as well as their optionality in the 25 studied strains of the FTSC clearly argue in favor of their ancestral origin in the *Fusarium* genus, accompanied by frequent events of gain and loss occurring during short evolution periods. Among the nine different introns characterized in the *F. tricinctum* and *F. avenaceum* mitochondrial genomes, three were present in all the 25 studied strains, and the other six were found optional. Among the latter, this optionality was inter-specific for *nad4L1iFave1* (present in all *F. avenaceum* strains and absent from all *F. tricinctum* strains) and *cox1 iFtri1* (present in all *F. tricinctum* strains and absent from all *F. avenaceum* strains). The presence/absence of these introns thus provide a clear discrimination of *F. tricinctum* and *F. avenaceum* species, among the 25 studied strains. The observed high percentages of nucleotide identity between orthologous intron sequences from phylogenetically distant species supports the hypothesis of intron gains after lateral transfers. Here, up to eight lateral transfers have been evidenced and concern the introns *nad4L iFave1*, *cob i1*, *cox1 iFave1*, *cox1 iFtri1*, *cox1 i2*, and *cox1 i3* (Table 2).

In most of the species of the fungal kingdom, the mitochondrial *cox1* gene is the richest in group I introns [22], sometimes leading to astonishing “long” genes containing up to 18 introns in the Basidiomycota *Agaricus bisporus*. In Ascomycota, “long genes” have also been described, especially in species belonging to the *Fusarium* genus [18] in the FSAMSC (up to 13 introns in *F. graminearum*) or in the basal FSSC (eight introns reported in *F. solani*). On the contrary, species of the FOSC do not possess any intron in their *cox1* gene [19]. These reports argue for a high mobility of the group I introns of the *cox1* gene, with frequent events of gain and loss during the evolution of the *Fusarium* genus. Our results support this high mobility of the *cox1* mitochondrial introns in the *Fusarium* genus, with evidences of lateral transfers between introns described in the FTSC and *F. mangiferae* (FFSC) for *cox1 iFave1*, and three introns of *F. solani* (FSSC) for *cox1 iFtri1*, *cox1 i2* and *cox1 i3*. The co-location of these three introns in the same 3′ region of *cox1* and the percentages of nt identity between orthologous sequences suggest that they could have been transferred at the same time in a single event. Moreover, for *cox1 iFtri1* and *cox1 i3*, a second horizontal transfer may also have occurred involving highly distant species, an ancestor of the Basidiomycota *Agrocybe agegerita* and of the Ascomycota *A. tubigensis*, respectively. Such lateral transfers between an Ascomycota and *A. aegerita* has previously been reported [37]. Considering the *nad4L iFave1*, our results suggest two hypotheses: (*i*) this *Fusarium* ancestral mobile genetic element may have been lost (as in *F. tricinctum*) then recently re-gained by an event of lateral transfer involving *F. avenaceum* and a species phylogenetically related to *T. asperellum,* or (*ii*) the ancestral *nad4L iFave1* intron may have been maintained and recently transferred to *T. asperellum* by a lateral transfer. In the absence of other Ascomycota orthologous sequence with high nucleotide identity with the *T. asperellum* intron sequence, the second hypothesis seems more likely. Similarly, the incongruent relationship between the *cob i1* intron sequences of *F. tricinctum* and *F. avenaceum* strains with the orthologous sequence reported in *F. venenatum* suggests that a lateral transfer of this mobile genetic element may have occurred between *F. venenatum* and an ancestor of *F. tricinctum* and *F. avenaceum*. Notably, since *cob i1* is present in all *F. avenaceum* studied strains but not in all *F. tricinctum* ones, this intron may have been lost during the most recent evolution leading to the clade here referred as group A of the *F. tricinctum* species.

Comparing intron sequence polymorphism between *F. tricinctum* INRA104 and *F. avenaceum* FaLH27, little variability was detected, i.e.*,* at levels comparable to those found in mitochondrial CDS sequences. Moreover, all the nine introns carried an intact *heg* encoding a potentially functional enzyme. Twenty years ago, Goddard and Burt [36], studying the behavior of the mitochondrial *ω-heg* and its host intron in 20 yeast species, established a cyclic model with three steps: invasion, erosion and finally loss, followed by re-invasion. Their data led to the estimation that each step is achieved in about two million years, and that a long-term maintenance of an intact *heg* requires frequent lateral transmission events. This model [36] was later confirmed and extended by Koufopanou et al. [38] who reported a high adaptation of a *heg* to its lateral transmission. The frequency of these lateral transmission events appears, consequently, to play a major role in the widespreading of mitochondrial group I introns and *heg*. According to this cyclic model of intron evolution, maintenance of a functional HE is required only during the invasion phase of these self-splicing mobile genetic elements. When the parasitic element is fixed in a population, the HE activity becomes unnecessary, submitted to neutral selection and can be randomly lost [36, 39]. However, numerous putatively functional HEs were also reported to be maintained over long evolutionary periods in populations of species considered as mostly asexual. Such findings and implications have been extensively discussed by Gogarten et al. [39]. In this paper, they list several hypotheses to explain the maintenance of a functional HE, such as, for example, (i) an unsuspected level of intra-specific (sexual) and/or inter-specific matings, (ii) a complex population structure where the mobile element is not fixed, or (iii) an unknown role of the HE which will be selected because increasing the fitness of its host. Here, all these hypotheses could also explain the high conservation and putative functionality observed with all the nine *hegs* and group I introns described in the *F. tricinctum* and *F. avenaceum* mitochondrial genomes, independently from the fact that these mobile genetic elements were found involved in lateral transfers (as it is the case for most of them) or not.

## Conclusions

*Fusarium tricinctum* and *Fusarium avenaceum* are two phylogenetically close phytopathogenic species, producers of “emerging” mycotoxins including enniatins and, thus, potentially involved in future food-safety crises. In this paper, a comparative analysis of the molecular organization of their mitochondrial genome has been carried out in a set of 25 wild strains representing both species. This analysis shows that both species can easily be differentiated by polymorphic mitochondrial sequences, such as Indels occurring in the Lv-uORF region.

Moreover, both genomes were shown to harbour optional (inter- or intra-specifically) group I introns, all carrying putatively functional *heg*s, arguing for a high mobility of these introns occurring during short evolution periods. The gain events of these selfish (parasitic) mobile elements were shown to involve, for most of them, lateral transfers between phylogenetically distant species, suggesting an important role of sexual but also interspecific mating in the populations of these phytopathogens.

This study has also revealed a new type of mobile genetic element constituted by a palindromic arrangement of single nucleotide (A) n and (T) n microsatellite sequences whose presence was related to occurrence of SNPs and Indels in the neighbouring regions. Such mobile elements could represent an important driving force of mitochondrial genome evolution in *Fusarium* species but also in a broader range of fungal species.

## Methods

### *Fusarium spp*. strains and culture conditions, sequences used for taxonomic identification and phylogenetic studies

All the *F. tricinctum* and *F. avenaceum* strains used in this study (Additional file 3: Table S3) were issued from subcultures and monosporal (conidial) isolation of strains harvested from the wild (from maize or cereal crops with various geographical origins but mostly from various French regions) and are conserved in the INRA UR1264 MycSA laboratory or in the Fungal Culture Collection of the International Centre of Microbial Resources (CIRM-CF), curated (Marseille, France). Mycelia were cultured on solid PDA medium (Difco). All strains were previously assigned to the *F. tricinctum* or *F. avenaceum* species by, first, a morphological characterization of the mycelial hyphae and their produced asexual (macroconidia) and/or vegetative (chlamydospores) mitospores (according to [40]), then by using, qPCR, with species-specific primers [41, 42]. The Accession numbers of the sequences obtained in this study and used to build phylogenetic trees are also indicated in Additional file 3: Table S3.

### Mitochondrial genome annotation

The complete mitochondrial genome sequences of *F. tricinctum* strain INRA104 and *F. avenaceum* strain FaLH27 were previously obtained [16, 17]. Their full annotation was performed and manually curated after comparison with sequences in GenBank and EMBL databases using BLASTn and BLASTp [43]. To identify protein coding genes, MtDNA sequences were searched for open reading frames (ORF) using the ORFfinder software (https://www.ncbi.nlm.nih.gov/orffinder/). GeSeq [44] was further used for the annotation of *F. tricinctum* INRA104 and *F. avenaceum* FaLH27 guided by the mitochondrial genomes of *F. verticillioides*, *F. solani*, and *F. graminearum* as annotated in MiToFun (http://mitofun.biol.uoa.gr/index.html). Predictions were then crossed and confirmed by multi-alignments of nucleic acids and predicted protein sequences performed using Clustal Omega [45] and MUSCLE [46]. Mitochondrial transfer RNA genes (tRNAs) were located using the tRNA Scan-SE [47]. Mitochondrial maps and corresponding Genbank files were generated using ApE (http://jorgensen.biology.utah.edu/wayned/ape/) and SnapGene software (from GSL Biotech; available at snapgene.com). Introns were first identified by Blast analysis. For each intron, its borders (5′ and 3′ ends) were established after deletion of the highly conserved exonic sequences of each mitochondrial CDS then confirmed by alignment with orthologous sequences. In the same way, the assignment to group I and subgroup were deduced from those given to their orthologous sequences, mainly in Al-Reedy et al. [18] or Ferandon et al. [22] for *cox1* introns.

### DNA extraction and PCR

Whole genomic DNA was extracted according to the *N-*cethyl-*NNN*-trimethyl ammonium bromide (CTAB) procedure described in Barroso et al. [48].

Primers were designed to amplify and sequence nuclear and mitochondrial regions used as taxonomic and phylogenetic markers (Additional file 3: Table S4). These couples were defined from the alignment of the *F. tricinctum* and *F. avenaceum* nuclear gene sequences *rpb1*, *rpb2*, and of a part of the mitochondrial Lv-uORF. Primers were also designed to amplify each intron found in the mitochondrial sequences of *F. tricinctum* strain INRA104 or *F. avenaceum* strain FaLH27 (Additional file 3: Table S4). PCR mixes contained between 10 to 100 ng of fungal DNA, 1 μM of both primers, 100 μΜ of each dNTP, 2 units of Go*Taq* DNA polymerase (Promega Corp., Madison, Wis, USA) in a final volume of 50 μL buffer. PCR conditions were: 95 °C for 5 min; 35 x (95 °C for 30 s; primer hybridization at the indicated temperature (Additional file 3: Table S4) for 30 s; elongation at 72 °C for 90 to 120 s); 72 °C for 5 min.

### DNA sequencing and sequence analysis

PCR products were sequenced, according to the Sanger method, on both strands by Genewiz (UK), with both forward and reverse primers used to obtain each PCR product.

GenBank Accession numbers of the sequenced PCR products are indicated in Additional file 3: Table S3. Sequence comparisons with GenBank and EMBL databases were performed with BLAST [43]. Multi-alignments of nucleic acids were performed with MUSCLE 3.8.31 [46] and subsequently cleaned with Gblocks 0.91b [49]. Phylogenetic trees were computed using MrBayes 3.2.7a [50, 51] and parameters (Additional file 3, Table S5) estimated with MEGA X [52]. Tree rendering was performed with FigTree 1.4.4, displaying posterior probabilities as branch supports obtained after 1,000,000 generations.

## Supplementary information

**Additional file 1: **GenBank formatted annotated sequence of the *F. tricinctum* strain INRA104 MtDNA.

**Additional file 2: **GenBank formatted annotated sequence of the *F. avenaceum* strain FaLH27 MtDNA.

**Additional file 3: Table S1.** SNP and indel occurrence in F. tricinctum vs. F. avenaceum mitochondrial genomes. **Table S2.** Details of SNPs and Indels between F. tricinctum vs. F. avenaceum mitochondrial genomes. **Table S3.** F. tricinctum and F. avenaceum strains and sequences (GenBank Accession N°) used in this study. **Table S4.** Primers and PCR conditions used in this study. **Table S5.** MrBayes input parameters estimated with MEGA X. **Figure S1**. Unrooted phylogenetic tree of Fusarium species and of distant or closely related Ascomycota species based on cox1 complete CDS sequence alignment. Posterior probabilities (Bayesian inference; 1,000,000 generations) are indicated in red. Labels show species names followed by the name and/or number of the strain and the GenBank accession number corresponding to the sequence used. Basidio = Basidiomycota; Asco = Ascomycota; FSSC = *Fusarium solani* Species Complex; FSAMSC = Fusarium sambucinum Species Complex; FTSC = Fusarium tricinctum Species Complex; FOSC = Fusarium oxysporum Species Complex; FFSC = Fusarium fujikuroi Species Complex. **Figure S2.** Nucleotide alignments of the interspecific polymorphic region between F. tricinctum and F. avenaceum of the mitochondrial large variable region containing the uORF for studied F. tricinctum and F. avenaceum strains.

**Additional file 4:Table S6.** Strains and sequences (GenBank Accession N°) used in this study

## Data Availability

All the sequences determined in this study have been deposited at GenBank (see text and Additional file 3 Table S3 for Accession numbers). All the sequences used for phylogenetic analyses have been retrieved from the GenBank. Their accession numbers are listed in additional file 4, Table S6.

## Abbreviations

Fave: *Fusarium avenaceum*
FFSC: *Fusarium fujikuroi* Species Complex
FHB: Fusarium Head Blight
FOSC: *Fusarium oxysporum* Species Complex
FSAMSC: *Fusarium sambucinum* Species Complex
FSSC: *Fusarium solani* Species Complex
Ftri: *Fusarium tricinctum*
FTSC: *Fusarium tricinctum* Species Complex
Indel: Insertion/deletion sequence
*heg*: homing endonuclease gene
LSU-rDNA: Large subunit ribosomal DNA
MtDNA: Mitochondrial genome
SNP: Single Nucleotide Polymorphism
SSU-rDNA: Small subunit rDNA
uORF: unidentified Open Reading Frame
