# Supplementary material for: Evolution of Fusarium tricinctum and Fusarium avenaceum mitochondrial genomes is driven by mobility of introns and of a new type of palindromic microsatellite repeats
Source: BMC Genomics. 2020 May 12;21:358. doi: 10.1186/s12864-020-6770-2 (PMC7218506; doi:10.1186/s12864-020-6770-2)
Supplement: Supplementary file 3 — Additional file 3: Table S1. SNP and indel occurrence in F. tricinctum vs. F. avenaceum mitochondrial genomes. Table S2. Details of SNPs and Indels between F. tricinctum vs. F. avenaceum mitochondrial genomes. Table S3. F. tricinctum and F. avenaceum strains and sequences (GenBank Accession N°) used in this study. Table S4. Primers and PCR conditions used in this study. Table S5. MrBayes input parameters estimated with MEGA X. Figure S1. Unrooted phylogenetic tree of Fusarium species and of distant or closely related Ascomycota species based on cox1 complete CDS sequence alignment. Posterior probabilities (Bayesian inference; 1,000,000 generations) are indicated in red. Labels show species names followed by the name and/or number of the strain and the GenBank accession number corresponding to the sequence used. Basidio = Basidiomycota; Asco = Ascomycota; FSSC = Fusarium solani Species Complex; FSAMSC = Fusarium sambucinum Species Complex; FTSC = Fusarium tricinctum Species Complex; FOSC = Fusarium oxysporum Species Complex; FFSC = Fusarium fujikuroi Species Complex. Figure S2. Nucleotide alignments of the interspecific polymorphic region between F. tricinctum and F. avenaceum of the mitochondrial large variable region containing the uORF for studied F. tricinctum and F. avenaceum strains. [file 12864_2020_6770_MOESM3_ESM.docx]

Additional tables and figures for:

**Evolution of *Fusarium tricinctum* and *Fusarium avenaceum* mitochondrial genomes is driven by mobility of introns and of a new type of palindromic microsatellite repeats**

**Authors:**

Nadia Ponts^1^, Charlotte Gautier^1^, Jérôme Gouzy^2^, Laetitia Pinson-Gadais^1^, Marie Foulongne-Oriol^1^, Christine Ducos^1^, Florence Richard-Forget^1^, Jean-Michel Savoie^1^, Chen Zhao^3^, Gérard Barroso^1,4*^

**Addresses:**

^1^INRAE, MycSA, F-33882 Villenave d’Ornon, France*

^2^LIPM, Université de Toulouse, INRAE, CNRS, Castanet-Tolosan, France

^3^Academy of National Food and Strategic Reserves Administration, Beijing, China

^4^Univ. Bordeaux, INRAE, MycSA, F-33882 Villenave d’Ornon, France

***Author for correspondence:** Gerard Barroso, University of Bordeaux, INRAE, MycSA, Villenave d’Ornon, France, tel: + 33 (0)5 57 12 25 95, [gerard.barroso@u-bordeaux.fr](mailto:gerard.barroso@u-bordeaux.fr)

16-digit ORCID: 0000-0002-4553-9988.

**Additional tables**

| **Table S1** SNP and indel occurrence in *F. tricinctum vs.* *F. avenaceum* mitochondrial genomes | | | | | | | | |
| --- | --- | --- | --- | --- | --- | --- | --- | --- |
| ***Locus*** | **Region size (bp) in *F. tricinctum*** | **Region size (bp) in *F. avenaceum*** | **SNP count** | **SNP/kb** | **Indel count** | **Indel sizes** | **Indel/kb** | **Total mutations /kb** |
|  |  |  |  |  |  |  |  |  |
| **In the whole mitochondrial genome** | | | | | | | | |
|  | **48,506** | **49,396** | **314** | **6.47** | **66** | **1.713** | **1.36** | **7.83** |
| **In rDNA or CDS** | | | | | | | | |
| **LSU** | 3,151 | 3,151 | 2 | 0.63 | 0 | 0 | 0 | 0.63 |
| **SSU-rDNA** | 1,616 | 1,616 | 2 | 1.24 | 0 | 0 | 0 | 1.24 |
| ***rps3*** | 1,440 | 1,440 | 3 | 2.08 | 0 | 0 | 0 | 2.08 |
| ***nad2*** | 1,665 | 1,665 | 1 | 0.60 | 0 | 0 | 0 | 0.60 |
| ***nad3*** | 414 | 414 | 1 | 2.42 | 0 | 0 | 0 | 2.42 |
| ***atp9*** | 225 | 225 | 2 | 8.89 | 0 | 0 | 0 | 8.89 |
| ***cox2*** | 750 | 750 | 1 | 1.33 | 0 | 0 | 0 | 1.33 |
| ***nad4L*** | 269 | 269 | 0 | 0 | 0 | 0 | 0 | 0 |
| ***nad5*** | 2,007 | 2,007 | 3 | 1.49 | 0 | 0 | 0 | 1.49 |
| ***cob*** | 1,173 | 1,173 | 3 | 2.56 | 0 | 0 | 0 | 2.56 |
| ***cox1*** | 1,593 | 1,593 | 3 | 1.88 | 0 | 0 | 0 | 1.88 |
| ***nad1*** | 1,110 | 1,110 | 2 | 1.80 | 0 | 0 | 0 | 1.80 |
| ***nad4*** | 1,458 | 1,458 | 2 | 1.37 | 0 | 0 | 0 | 1.37 |
| ***atp8*** | 147 | 147 | 1 | 6.80 | 0 | 0 | 0 | 6.80 |
| ***atp6*** | 801 | 801 | 1 | 1.25 | 0 | 0 | 0 | 1.25 |
| ***cox3*** | 810 | 810 | 3 | 3.70 | 0 | 0 | 0 | 3.70 |
| ***nad6*** | 699 | 699 | 0 | 0 | 0 | 0 | 0 | 0 |
| ***26 tRNA*** | 1,921 | 1,921 | 0 | 0 | 0 | 0 | 0 | 0 |
| **Total** | **21,249** | **21,249** | **30** | **1.41** | **0** | **0** | **0** | **1.41** |
| **In intergenic sequences** | | | | | | | | |
| **intergenic 13** | 245 | 245 | 2 | 8.16 | 0 | 0 | 0 | 8.16 |
| **intergenic 14** | 523 | 514 | 12 | 22.94 | 1 | 16 | 1.91 | 24.86 |
| **intergenic 15** | 211 | 246 | 3 | 14.22 | 1 | 35 | 4.74 | 18.96 |
| **intergenic 16** | 457 | 476 | 10 | 21.88 | 2 | 25 | 4.38 | 26.26 |
| **intergenic 17** | 286 | 308 | 0 | 0 | 1 | 22 | 3.50 | 3.50 |
| **intergenic 18** | 926 | 1,018 | 16 | 17.28 | 3 | 96 | 3.24 | 20.52 |
| **intergenic 19** | 161 | 167 | 8 | 49.69 | 1 | 11 | 6.21 | 55.90 |
| **intergenic 20** | 1,876 | 1,838 | 24 | 12.79 | 9 | 302 | 4.80 | 17.59 |
| **intergenic 21** | 328 | 326 | 8 | 24.39 | 1 | 2 | 3.05 | 27.44 |
| **intergenic 22** | 1,221 | 1,323 | 7 | 5.73 | 3 | 116 | 2.46 | 8.19 |
| **intergenic 23** | 652 | 635 | 7 | 10.74 | 2 | 29 | 3.07 | 13.80 |
| **intergenic 24** | 137 | 136 | 0 | 0 | 1 | 1 | 7.30 | 7.30 |
| **intergenic 25** | 191 | 193 | 3 | 15.71 | 0 | 0 | 0 | 15.71 |
| **intergenic 26** | 562 | 565 | 6 | 10.68 | 3 | 173 | 5.34 | 16.01 |
| **intergenic 27** | 343 | 319 | 0 | 0 | 2 | 40 | 5.83 | 5.83 |
| **intergenic 28** | 201 | 189 | 4 | 19.90 | 2 | 40 | 9.95 | 29.85 |
| **intergenic 29** | 230 | 198 | 3 | 13.04 | 3 | 39 | 13.04 | 26.09 |
| **intergenic 30** | 45 | 45 | 0 | 0 | 0 | 0 | 0 | 0 |
| **intergenic 31** | 524 | 521 | 11 | 20.99 | 2 | 9 | 3.82 | 24.81 |
| **intergenic 32** | 980 | 990 | 5 | 5.10 | 7 | 204 | 7.14 | 12.24 |
| **intergenic 33** | 189 | 189 | 2 | 10.58 | 0 | 0 | 0 | 10.58 |
| **intergenic 34** | 1 | 1 | 0 | 0 | 0 | 0 | 0 | 0 |
| **intergenic 35** | 149 | 149 | 5 | 33.56 | 0 | 0 | 0 | 33.56 |
| **intergenic 36** | 299 | 314 | 1 | 3.34 | 1 | 15 | 3.34 | 6.69 |
| **Total** | **10,737** | **10,905** | **137** | **12.76** | **45** | **1,175** | **4.19** | **16.95** |
| **In intronic sequences (present in both species)** | | | | | | | | |
| **Intron IA LSU** | 698 | 698 | 4 | 5.73 | 3 | 82 | 4.30 | 10.03 |
| **Intron IB *cox2*** | 1,126 | 1,135 | 0 | 0 | 1 | 11 | 0.89 | 0.89 |
| **Intron ID *cob*** | 1,232 | 1,232 | 0 | 0 | 0 |  | 0 | 0 |
| **Intron I *cox1* *i2*** | 1,326 | 1,317 | 5 | 3.77 | 1 | 10 | 0.75 | 4.52 |
| **Intron I *cox1 i3*** | 1,092 | 1,092 | 3 | 2.75 | 0 |  | 0 | 2.75 |
| **Intron I *cox1 i4*** | 1,018 | 1,018 | 2 | 1.96 | 0 |  | 0 | 1.96 |
| **Total** | **6,492** | **6,492** | **14** | **1,63** | **5** | **103** | **0.58** | **2.21** |
| **In the Large variable region** | | | | | | | | |
| **Upstream tRNA cluster (inter. 1-5)** | 439 | 438 | 26 | 59.23 | 1 | 1 | 2.28 | 61.50 |
| **uORF (+ flanking regions)** | 6,320 | 6,276 | 88 | 13.92 | 6 | 57 | 0.95 | 14.87 |
| **Downstream tRNA cluster (inter. 6-12)** | 1,182 | 1,293 | 19 | 16.07 | 9 | 377 | 7.61 | 23.69 |
| **Total** | **7,941** | **8,007** | **133** | **16.75** | **16** | **435** | **2.01** | **18.76** |

| **Table S2** Details of SNPs and Indels between *F. tricinctum* *vs.* *F. avenaceum* mitochondrial genomes | | |
| --- | --- | --- |
| **Location** | **SNPs*** | **Indels**** |
| **In rDNA or CDS** | | |
| LSU | C/G, A/T |  |
| SSU-rDNA | G/A, T/C |  |
| *rps3* | A/G, G/A, T/C |  |
| *nad2* | T/C |  |
| *nad3* | T/A |  |
| *atp9* | G/A, A/G |  |
| *cox2* | T/C |  |
| *nad4L* |  |  |
| *nad5* | T/C, T/A, C/T |  |
| *cob* | A/T, C/T, T/C |  |
| *cox1* | C/T, T/C, A/T |  |
| *nad1* | A/G, G/A |  |
| *nad4* | T/C, T/A |  |
| *atp8* | C/T |  |
| *atp6* | C/T |  |
| *cox3* | A/G, C/T, A/T |  |
| *nad6* |  |  |
| *26 x tRNA* |  |  |
| **In intergenic sequences** | | |
| intergenic 13 | G/A, C/T |  |
| intergenic 14 | 4 C/T, C/A, A/T, 3 G/A, 3 A /G | Ins.9; Ins.7 |
| intergenic 15 | T/A, C/G, G/C | Del.35 |
| intergenic 16 | A/C, C/T, G/C, 4T/C, A/T, A/G, G/A | Del.22; Ins.3 |
| intergenic 17 |  | Del.22 |
| intergenic 18 | 3 A/G, 2 T/C, T/G, 2 G/C, 2 C/T, G/A, 2 T/A | Del.92; Del.2; Ins.2; A-Ins.1/T-Del.1 |
| intergenic 19 | 2 C/T, A/G, G/A | Del.6; Del.5; A-Del.2/T-Ins.2 |
| intergenic 20 | 3 T/A, 2 G/A, 4 T/C, 5 C/T, 3 A/G, 1 T/G, 1C/G, 1 A/C, 1 A/T | Ins.16; Ins.4; Del.3; Del.8; Del.15; Del.2; Del.103; Del.1; Ins.114; Ins.23; Ins.3; Ins.7; Ins.3; A-Ins.1/T-Ins.1  T-Ins.1 |
| intergenic 21 | 2 T/A, T/G, G/A, T/C, G/T, A/C, G/C | Ins.2 |
| intergenic 22 | 2 T/G, C/G, A/G, A/T, 2 C/T | Ins.7; Del.36; Del.73 |
| intergenic 23 | C/T, G/A, A/T | Del.5; Ins.24; A-Ins.1/T-Del.1 |
| intergenic 24 |  | Ins.1 |
| intergenic 25 | G/T | A-Del.1/T-Del.1 |
| intergenic 26 | 3 G/A | Del.4; Del.139; Del.20; Ins.7; Ins.3;  A-Del.1/T-Ins.1/ T-Ins.1 |
| intergenic 27 |  | Del.8; Ins.32 |
| intergenic 28 | G/C, C/T, T/A, G/C | Del.14; Ins.26 |
| intergenic 29 | 3 C/T | Ins.9; Ins.3; Ins.4; Ins.6; Ins.17 |
| intergenic 30 |  |  |
| intergenic 31 | A/T, 2 T/C, A/G, G/A, G/T, T/G | Ins.5; Del.4; A-Ins.3/T-Del.1 |
| intergenic 32 | A/G, 2 G/A, G/T, T/A | Ins.8; Ins.4; Ins.8; Ins.3; Ins.7; Ins.3; Ins.6; Ins.5; Ins.8; Ins.3; Del.81; Del.34; Ins.34 |
| intergenic 33 | G/A, T/C |  |
| intergenic 34 |  |  |
| intergenic 35 | C/T, T/G, G/A, A/C, G/T |  |
| intergenic 36 | T/A | Del.15 |
| **In intronic sequences** | | |
| Intron IA LSU | T/G, G/T, A/C, T/A | Del.43; Del.9; Del.4; Ins.23; Ins.2 |
| Intron IB *cox2* |  | Del.9; Del.2 |
| Intron ID *cob* |  |  |
| Intron I *cox1 i2* | 2 T/G, A/T, 2 A/G | Ins.8; Ins.2 |
| Intron I *cox1 i3* | 2 G/A, T/C |  |
| Intron I *cox1 i4* | 2 T/G |  |
| **In the large variable region** | | |
| tRNA cluster upstream (intergen. 1-5) | A/C, C/A, G/A, T/G | Ins.1; A-Del.1/T-Ins.1 |
| uORF (+ flanking regions) | G5-4, A7-6, A5-6, G6-5, 8 A/T, 17 A/G, 5 A/C, 3T/A, 2T/G, 14T/C, 13G/A, 3G/T, 6G/C, 3C/A, 5C/T, 6C/G | Del.3; Ins.15; Ins.2; Ins.24; Ins.4; Del.1; Del.2; Ins.6 |
| tRNA cluster downstream (intergen. 6-12) | T/A, 3 T/C, 3 A/T, 3 A/G, G/T, G/A, G/C, 3 C/T, 2 C/A, C/G | Ins.108; Ins.1; Ins.8; Del.17; Del.5; Ins.1; Ins.8; Del.17; Del.5; Ins.1; Del.4; Del.18; Del.42 ; A-Del.1 |

| **Table S3** *F. tricinctum* and *F. avenaceum* strains and sequences (GenBank Accession N°) used in this study | | | | | | |
| --- | --- | --- | --- | --- | --- | --- |
| **Species** | **Specimen-voucher** | **Repository** | **GenBank Accession Numbers** | | | **Reference** |
|  |  |  | ***rpb1*** | ***rpb2*** | ***Fusarium* mit Hypothetical protein** |  |
| *F. tricinctum* | INRA 104^1^ | MycSA coll./CIRM-CF | QFZF01000000 | QFZF01000000 | MH667507 | This work, Ponts et al. 2018 |
| *F. tricinctum* | INRA 105 | MycSA coll./CIRM-CF | MH667531 | MH667565 | MH667498 | This work |
| *F. tricinctum* | INRA 106 | MycSA coll./CIRM-CF | MH667532 | MH667566 | MH667499 | This work |
| *F. tricinctum* | INRA 610 | MycSA collection | MH667542 | MH667563 | MH667516 | This work |
| *F. tricinctum* | INRA 521 | MycSA collection | MH667533 | MH667554 | MH667519 | This work |
| *F. tricinctum* | INRA 522 | MycSA collection | MH667534 | MH667555 | MH667508 | This work |
| *F. tricinctum* | INRA 523 | MycSA collection | MH667535 | MH667556 | MH667509 | This work |
| *F. tricinctum* | INRA 524 | MycSA collection | MH667536 | MH667557 | MH667510 | This work |
| *F. tricinctum* | INRA 525 | MycSA collection | MH667537 | MH667558 | MH667511 | This work |
| *F. tricinctum* | INRA 526 | MycSA collection | MH667538 | MH667559 | MH667512 | This work |
| *F. tricinctum* | INRA 527 | MycSA collection | MH667539 | MH667560 | MH667513 | This work |
| *F. tricinctum* | INRA 528 | MycSA collection | MH667540 | MH667561 | MH667514 | This work |
| *F. tricinctum* | INRA 529 | MycSA collection | MH667541 | MH667562 | MH667515 | This work |
| *F. tricinctum* | MUCL 18522 | BCCM/MUCL | MH667530 | MH667564 | ND | This work |
| *F. avenaceum* | INRA 112 | MycSA coll./CIRM-CF | MH667521 | MH667546 | MH667500 | This work |
| *F. avenaceum* | INRA 494 | MycSA collection | MH667522 | MH667548 | MH667501 | This work |
| *F. avenaceum* | INRA 495 | MycSA collection | MH667523 | MH667549 | MH667502 | This work |
| *F. avenaceum* | INRA 496 | MycSA collection | MH667524 | MH667550 | MH667503 | This work |
| *F. avenaceum* | INRA 497 | MycSA collection | MH667525 | MH667551 | MH667504 | This work |
| *F. avenaceum* | INRA 498 | MycSA collection | MH667526 | MH667552 | MH667505 | This work |
| *F. avenaceum* | INRA 499 | MycSA collection | MH667527 | MH667553 | MH667506 | This work |
| *F. avenaceum* | INRA 611 | MycSA collection | MH667528 | MH667543 | MH667517 | This work |
| *F. avenaceum* | INRA 612 | MycSA collection | MH667529 | MH667544 | ND | This work |
| *F. avenaceum* | CBS 143.25 | Westerdijk Fungal Biodiversity Institute | MH667520 | MH667545 | MH667497 | This work |
| *F. avenaceum* | FaLH27 (DAOM242378)^2^ | Canadian Collection of Fungal Cultures | JQGE01000019 | JQGE01000018 | JQGE01000002 | Lysoe et al. 2014 |
| ND = not determined; ^1^Accession number for mitochondrial genome sequence CM009895; ^2^Accession number for mitochondrial genome sequence JQGE01000002 | | | | | | |

| **Table S4** Primers and PCR conditions used in this study | | | | | |
| --- | --- | --- | --- | --- | --- |
| **Primer name** | **Sequence 5'-3'** | **Target (product size in bp)*** | **Primers Positions** (nt)** | **PCR conditions** | |
|  |  |  |  | **Ta (°C)** | **Elongation time (sec)** |
| **Table 2*A*.** Primers for taxonomic and phylogenetic studies | | | | | |
| RPB1U | GCACCCTGCCTCATTTCAC | nuclear *rpb1* | 2,402–2,420 | 54 | 90 |
| RPB1R | CAACCATTTCACCAGGGTTC | (852 / 5280) | 3,253–3234 | 54 | 90 |
|  |  |  |  |  |  |
| RPB2U | CAACACCCCCATCGGAC | nuclear *rpb2* | 1,560–1,576 | 54 | 90 |
| RPB2R | ACCAAGAATCATACTGGGATG | (820 / 3810) | 2,379–2,359 | 54 | 90 |
|  |  |  |  |  |  |
| uORF D U | GGCTAAGATGTAATTTTATTGC | mit uORF | 6,430–6,451 | 45 | 90 |
| uORF D R | ATAATAATCTTCATCTTCTATAGG | (995) | 7,424–7,404 | 45 | 90 |
|  |  |  |  |  |  |
| **Table 2*B***. Primers for mit intronic sequences | | | | | |
| UFgi1 | ATATAACAGTGTAATTACAGC | *cox1 iFave i1* (1,778) | 29,131–29,151 | 50 | 120 |
| RFgi3 | CCACCGGCTACTTCAAAG | or *Ftri* exon (516) | 29,646–29,629 | 50 | 120 |
|  |  |  |  |  |  |
| UFgi456 | CTTTGAAGTAGCCGGTGG | *cox1 iFtri i1* (1,256) | 29,629–29,646 | 50 | 120 |
| RFg7 | ATCCTAGTATTCCTATAGAC | or *Fave* exon (194) | 30,884–30,865 | 50 | 120 |
|  |  |  |  |  |  |
| UFgi10 tri | ATTTATGTTTACAATAGGAGGG | *cox1 i2* | 31,066–31,086 | 50 | 120 |
| RFgi10 tri | CTACATAGTAAGTATCGTGG | (1,412 / 1,403) | 32,477–32,458 | 50 | 120 |
|  |  |  |  |  |  |
| UFgi11 | CCACGATACTTACTATGTAG | *cox1 i3* | 32,458–32,477 | 54 | 120 |
| Rfgi11 | GCAAACATTGCGAATACAG | (1,164) | 33,621–33,603 | 54 | 120 |
|  |  |  |  |  |  |
| UFgi12 | CTGTATTCGCAATGTTTGC | *cox1 i4* | 33,603–33,621 | 54 | 120 |
| RFgi12 | CCTTGTAAACCTAAGAAATG | (1,169) | 34,771–34,752 | 54 | 120 |
|  |  |  |  |  |  |
| cox2 tri U | GAACTTCACGATAATATTATGTAC | *cox2 i1* | 17,351–17,369 | 50 | 120 |
| cox2 tri R | GAAGGGTCATTAACTTCATCC | (1,362 / 1,371) | 18,712–18,692 | 50 | 120 |
|  |  |  |  |  |  |
| NAD5 tri U | TTGGTGCAATGGCTAAAAGTTCT | *nad5 iFtri* (1,237) | 21,145–21,167 | 54 | 120 |
| NAD5 tri R | AGCTAAACACAGTTGTAGTTGCAC | or *Fave* exon (212) | 22,383–22,359 | 54 | 120 |
|  |  |  |  |  |  |
| NAD4Ltri U | TAGTAGTTGCTGGTGCAGAATCTGC | *nad4L iFtri (*1,656) | 20,402–20,426 | 54 | 120 |
| NAD4Ltri R | GCTACCTGTAACACCGACTTTTC | or *Fave* exon (175) | 20,576–20,554 | 54 | 120 |
|  |  |  |  |  |  |
| cob tri U | ACGCATCATATAGAGCACCAAG | *cob i1* | 24758–24779 | 54 | 120 |
| cob tri R | GTCCAATTCATGGAACAGCACTA | (1,400) | 26157–26139 | 54 | 120 |
| *size of the amplified region in *F. tricinctum/F. avenaceum* reference genomes, in bp; **in *F. tricinctum* (1^st^ line) or *F. avenaceum* (2^nd^ line) nuclear gene or mtDNA | | | | | |

| **Table S5** MrBayes input parameters estimated with MEGA X | |
| --- | --- |
| **Sequences to analyze** | **Model predicted as best by MEGA X*** |
|  |  |
| All mitochondrial CDS | GTR+G+I |
| *cox1* (exonic) | GTR+G+I |
| *cob i1* | HKY+G |
| *cox1 i2* | T92 |
| *cox1 i3* | T92+I |
| *cox1 i4* | HKY |
| *cox1 iFave1* | T92+G |
| *cox1 iFtri1* | T92+G |
| *Cox2 i1* | T92 |
| *Nad4L iFave1* | T92 |
| Lv-uORF | T92 |
| *rpb1* | K2 |
| *rpb2* | K2+G |
| *Based on the lowest Bayesian Information Criterion | |

**Additional figures**

**Figure S1.** Unrooted phylogenetic tree of *Fusarium* species and of distant or closely related *Ascomycota* species based on *cox1* complete CDS sequence alignment. Posterior probabilities (Bayesian inference; 1,000,000 generations) are indicated in red. Labels show species names followed by the name and/or number of the strain and the GenBank accession number corresponding to the sequence used. Basidio = Basidiomycota; Asco = Ascomycota; FSSC = *Fusarium solani* Species Complex; FSAMSC = *Fusarium sambucinum* Species Complex; FTSC = *Fusarium tricinctum* Species Complex; FOSC = *Fusarium oxysporum* Species Complex; FFSC = *Fusarium fujikuroi* Species Complex.


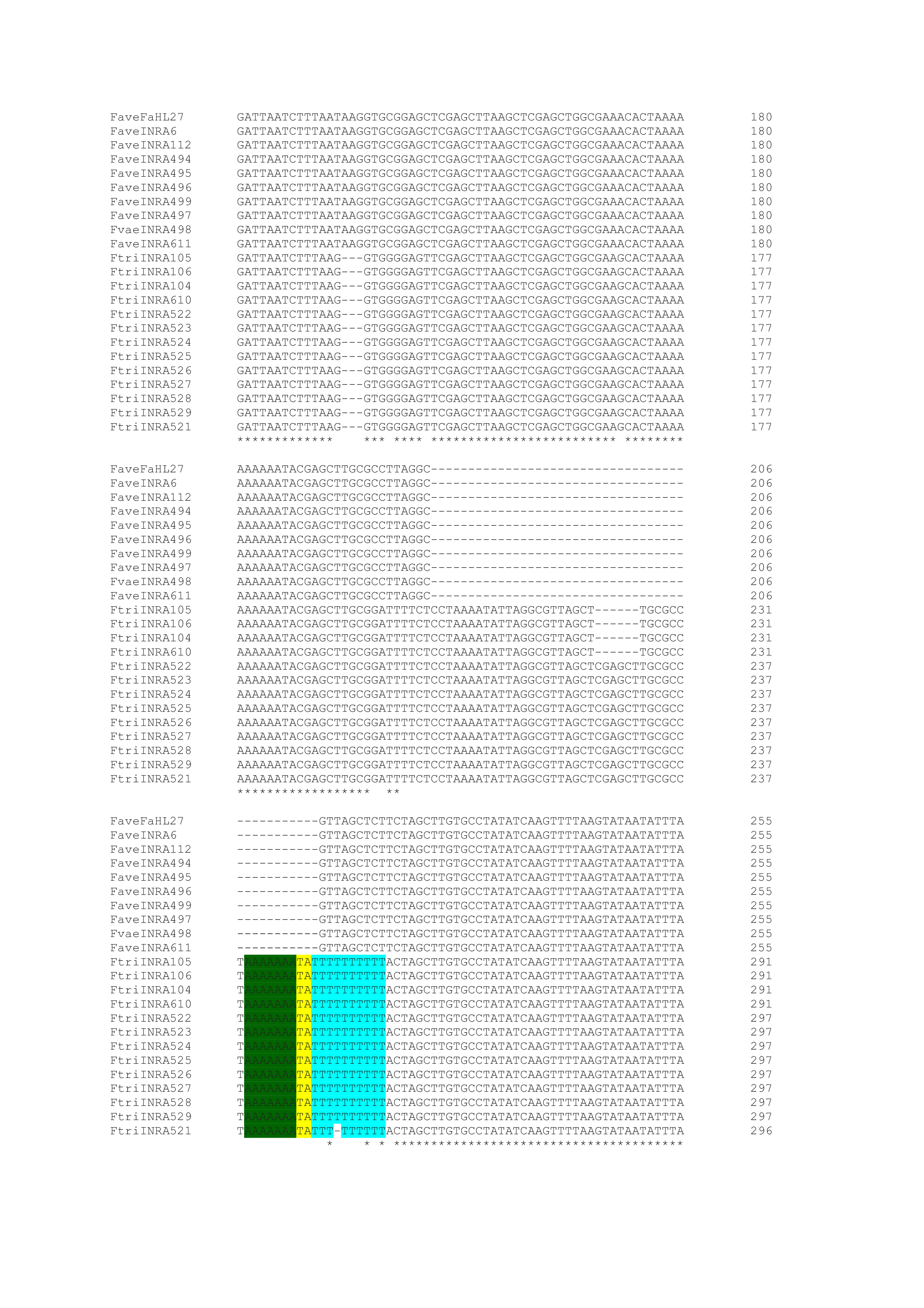


**Figure S2.** Nucleotide alignments of the interspecific polymorphic region between *F. tricinctum* and *F. avenaceum* of the mitochondrial large variable region containing the uORF for studied *F. tricinctum* and *F. avenaceum* strains.
