# Supplementary material for: Evolution of Fusarium tricinctum and Fusarium avenaceum mitochondrial genomes is driven by mobility of introns and of a new type of palindromic microsatellite repeats
Source: BMC Genomics. 2020 May 12;21:358. doi: 10.1186/s12864-020-6770-2 (PMC7218506; doi:10.1186/s12864-020-6770-2)
Supplement: Supplementary file 4 — Additional file 4:Table S6. Strains and sequences (GenBank Accession N°) used in this study [file 12864_2020_6770_MOESM4_ESM.docx]

| **Table S6** Strains and sequences (GenBank Accession N°) used in this study | | | | |  |  |
| --- | --- | --- | --- | --- | --- | --- |
| **Species** | **Specimen-voucher** | **GenBank Accession Numbers** | | |  |  |
|  |  | ***rpb1*** | ***rpb2*** | **mitochondrial genome** |  |  |
| *Agaricus bisporus* | H97 |  |  | JX271275 |  |  |
| *Agrocybe aegerita* | SM47 |  |  | AF010257 |  |  |
| *Aspergillus tubingensis* | 932 |  |  | NC007597 |  |  |
| *Beauveria bassiana* | K4 |  |  | KT201148 |  |  |
| *Bopolaris cookei* | LSLP18 |  |  | MF784482 |  |  |
| *Epichloe typhina* | E8 |  |  | KX066185 |  |  |
| *Fusarium acuminatum* | F201136 |  | KM520372 |  |  |  |
| *Fusarium acuminatum* | NRRL28449 | MG282373 | MG282402 |  |  |  |
| *Fusarium acuminatum* | NRRL28652 | MG282384 | MG282414 |  |  |  |
| *Fusarium acuminatum* | NRRL45994 | KC808323 |  |  |  |  |
| *Fusarium acuminatum* | NRRL52789 | JF741010 |  |  |  |  |
| *Fusarium acuminatum* | NRRL54210 |  | HM068328 |  |  |  |
| *Fusarium acuminatum* | NRRL54211 |  | HM068329 |  |  |  |
| *Fusarium acuminatum* | NRRL54212 |  | HM068330 |  |  |  |
| *Fusarium acuminatum* | NRRL54213 |  | HM068331 |  |  |  |
| *Fusarium acuminatum* | NRRL54216 |  | HM068334 |  |  |  |
| *Fusarium acuminatum* | NRRL54217 |  | HM068335 |  |  |  |
| *Fusarium acuminatum* | PUF035 |  | HQ423229 |  |  |  |
| *Fusarium acuminatum* | PUF036 |  | HQ423230 |  |  |  |
| *Fusarium acuminatum* | Tub05 |  | LT970738 |  |  |  |
| *Fusarium arthrosporoides* | NRRL26416 | MG282383 | MG282413 |  |  |  |
| *Fusarium avenaceum* | BBA64151 |  | HQ728167 |  |  |  |
| *Fusarium avenaceum* | BRIP64451 |  | KX058560 |  |  |  |
| *Fusarium avenaceum* | FaLH27 | JQGE01000019 | JQGE01000018 | JQGE01000002 |  |  |
| *Fusarium avenaceum* | FRCR09495 |  | GQ915486 |  |  |  |
| *Fusarium avenaceum* | MRC2195 |  | MH582372 |  |  |  |
| *Fusarium avenaceum* | NRRL25128 |  | MH582355 |  |  |  |
| *Fusarium avenaceum* | NRRL26911 | MG282372 | MG282401 |  |  |  |
| *Fusarium avenaceum* | NRRL36252 |  | MH582360 |  |  |  |
| *Fusarium avenaceum* | NRRL36374 | MG282366 | MG282395 |  |  |  |
| *Fusarium avenaceum* | NRRL54939 | JX171551 | JX171663 |  |  |  |
| *Fusarium avenaceum* | PUF034 |  | HQ423228 |  |  |  |
| *Fusarium circinatum* | MRC7870 |  |  | JX910419 |  |  |
| *Fusarium culmorum* | CBS139512 |  |  | KP827647 |  |  |
| *Fusarium flocciferum* | NRRL25473 | JX171514 | MH582356 |  |  |  |
| *Fusarium flocciferum* | NRRL45999 | HM347195 | MH582362 |  |  |  |
| *Fusarium flocciferum* | NRRL52714 |  | MH582363 |  |  |  |
| *Fusarium flocciferum* | NRRL52933 |  | MH582368 |  |  |  |
| *Fusarium flocciferum* | NRRL54147 |  | MH582369 |  |  |  |
| *Fusarium fujikuroi* | IM158289 |  |  | JX910420 |  |  |
| *Fusarium gerlachii* | CBS123666 |  |  | KM486533 |  |  |
| *Fusarium graminearum* | PH1 |  |  | DQ364632 |  |  |
| *Fusarium mangiferae* |  |  |  | KP742838 |  |  |
| *Fusarium oxysporum* | UASWSAC1 |  |  | KR952337 |  |  |
| *Fusarium petersiae* | JN14004 | MG386138 | MG386149 |  |  |  |
| *Fusarium petersiae* | JN14005 | MG386139 | MG386150 |  |  |  |
| *Fusarium poae* | 2516 |  |  | LYXU01000005 |  |  |
| *Fusarium sambucinum* | F4 |  |  | LSRD01000288 |  |  |
| *Fusarium solani* | VI |  |  | JN041209 |  |  |
| *Fusarium temperatum* |  |  |  | KP742837 |  |  |
| *Fusarium torulosum* | NRRL22748 | JX171502 | MH582376 |  |  |  |
| *Fusarium torulosum* | NRRL52772 | JF741003 | MH582377 |  |  |  |
| *Fusarium tricinctum* | INRA 104 | QFZF01000000 | QFZF01000000 | CM009895 |  |  |
| *Fusarium tricinctum* | NRRL25481 | JX171516 | JX171629 |  |  |  |
| *Fusarium tricinctum* | RBG4000 |  | HQ646398 |  |  |  |
| *Fusarium venenatum* | A3/5 |  |  | NW020311997 |  |  |
| *Fusarium verticilioides* | 7600 |  |  | JN041210 |  |  |
| *Agaricus bisporus* | H97 |  |  | JX271275 |  |  |
| *Agrocybe aegerita* | SM47 |  |  | AF010257 |  |  |
| *Aspergillus tubingensis* | 932 |  |  | NC007597 |  |  |
| *Beauveria bassiana* | K4 |  |  | KT201148 |  |  |
| *Bopolaris cookei* | LSLP18 |  |  | MF784482 |  |  |
| *Epichloe typhina* | E8 |  |  | KX066185 |  |  |
| *Hirsutella minnesotensis* | 3608 |  |  | KR139916 |  |  |
| *Hypomyces aurantius* |  |  |  | KU666552 |  |  |
| *Nectria cinnabarina* | 5151 |  |  | KT731105 |  |  |
| *Trichoderma asperellum* | B05 |  |  | NC037075 |  |  |
|  |  |  |  |  |  |  |
|  |  |  |  |  |  |  |
